# Anabaenolysins, Novel Cytolytic Lipopeptides from Benthic *Anabaena* Cyanobacteria

**DOI:** 10.1371/journal.pone.0041222

**Published:** 2012-07-19

**Authors:** Jouni Jokela, Linn Oftedal, Lars Herfindal, Perttu Permi, Matti Wahlsten, Stein Ove Døskeland, Kaarina Sivonen

**Affiliations:** 1 Division of Microbiology, Department of Food and Environmental Sciences, University of Helsinki, Helsinki, Finland; 2 Department of Biomedicine, University of Bergen, Bergen, Norway; 3 Translational Signaling Group, Haukeland University Hospital, Bergen, Norway; 4 Program in Structural Biology and Biophysics, Institute of Biotechnology, University of Helsinki, Helsinki, Finland; Institut Pasteur Paris, France

## Abstract

Two novel cyclic lipopeptides, anabaenolysin A and anabaenolysin B, were isolated from two benthic cyanobacterial strains of the genus *Anabaena*. This novel class of cyanobacterial lipopeptides has a general structure of a small peptide ring consisting of four amino acids from which two are proteinogenic and two unusual; glycine^1^, glycine^2^, 2-(3-amino-5-oxytetrahydrofuran-2-yl)-2-hydroxyacetic acid^3^ and a long unsaturated C_18_ β-amino acid^4^ with a conjugated triene structure. They are distinguished by the presence of a conjugated dienic structure in the C18 β-amino acid present in anabaenolysin A but not in anabaenolysin B. Conjugated triene structure generates a typical UV spectrum for anabaenolysins for easy recognition. Anabaenolysin A constituted up to 400 ppm of the cyanobacterial dry weight. We found evidence of thirteen variants of anabaenolysins in one cyanobacterial strain. This suggests that the anabaenolysins are an important class of secondary metabolites in benthic *Anabaena* cyanobacteria. Both anabaenolysin A and B had cytolytic activity on a number of mammalian cell lines.

## Introduction

Cyanobacteria produce an impressive variety of bioactive compounds from toxins to drug leads [1], [2], [3], [4]. Many of the bioactive compounds from cyanobacteria are cyclic peptides, the most known being the hepatotoxins microcystins and nodularins, which inhibit mammalian protein phosphatases [5] causing apoptosis [6]. Common for several cyanobacterial cyclic peptides are that they contain non-proteinogenic structures, such as the 3-amino-9-methoxy-2,6,8-trimethyl-10-phenyl-4,6-decadienoic acid (Adda) found in microcystins and nodularins, hydroxy acids like 2,2-dimethyl-3-hydroxyhexanoic acid (Dmhha) in the depsipeptide Palmyramide A [7] and imino bonds instead of amino bonds in the nostocyclopeptides [8], [9]. In lipopeptides, one or more amino acids are linked to fatty acid derivatives. They can be anything from small linear single amino acid derivative peptides with short carbon chain such as the spiroidesin from *Anabaena* cyanobacteria [10] to large cyclic peptides with long carbon chains attached, such as hassallidin from a *Hassallia* cyanobacterium [11]. These amphiphilic compounds display a wide range of bioactivities [1]. Most of the lipopeptides isolated from cyanobacteria are cytotoxic, but lipopeptides with anticancer, antibacterial, antifungal and other activities have also been isolated from cyanobacteria [1], [2], [4], [10], [11], [12].

We have previously shown that benthic cyanobacteria collected from brackish waters are a prolific source for cell death inducing compounds [13]–[14], [15]. In this study we describe the structure and lytic nature of two novel cytolytic cyclic lipopeptides, anabaenolysin A and B, isolated from brackish water benthic cyanobacteria of the genera *Anabaena*. The structural variability of anabaenolysins is elucidated from one of the *Anabaena* strains.

## Results and Discussion

### Detection and Purification of Cytolytic Activities in the *Anabaena* Cyanobacteria

**Figure 1:**
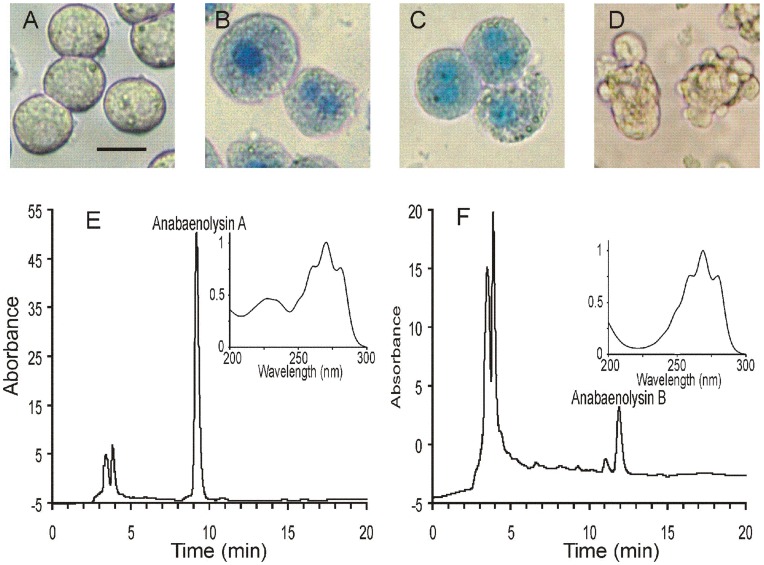
Bioassay-guided isolation of the cytolytic compounds anabaenolysin A (1) and B (2) from *Anabaena sp*. A–D: Extracts of *Anabaena* strains caused an influx of trypan blue into primary rat hepatocytes. Photomicrographs of rat hepatocytes incubated with solvent (A), aqueous methanol extracts of *Anabaena* strain XPORK 15F (B), XSPORK 27C (C) or a planktonic microcystin-containing *Anabaena* strain 318 (D), in the presence of the viability stain trypan blue. E and F: HPLC chromatogram of the bioactive solid phase extracts from XPORK 15F (E) and XSPORK 27C (F). Peaks were collected and tested for cytotoxicity on IPC-81 leukemia cells and all the cell death inducing activity eluted at 9.19 min (E) or 11.92 min (F). The inserts in (E) and (F) show absorbance spectra of **1** and **2**. Scale bar in A–D represents 20 µm.

We noted that primary rat hepatocytes treated with the aqueous methanol extract of the benthic *Anabaena* strains XPORK 15F and XSPORK 27C isolated from the Finnish south cost of Baltic Sea (Gulf of Finland) failed to exclude the dye trypan blue (Fig.****1B and C). Trypan blue is used to study intactness of outer cell membranes, and internalization shows that the membrane is permeabilized, and the cells are usually in a necrotic state. In comparison viable cells (Fig.****1A), or cells undergoing pure apoptotic death, such as hepatocytes treated with an extract containing the cyanobacterial toxin microcystin from a planktonic *Anabaena* strain 318 (Fig. 1D) did not show increased influx of trypan blue.

**Figure 2:**
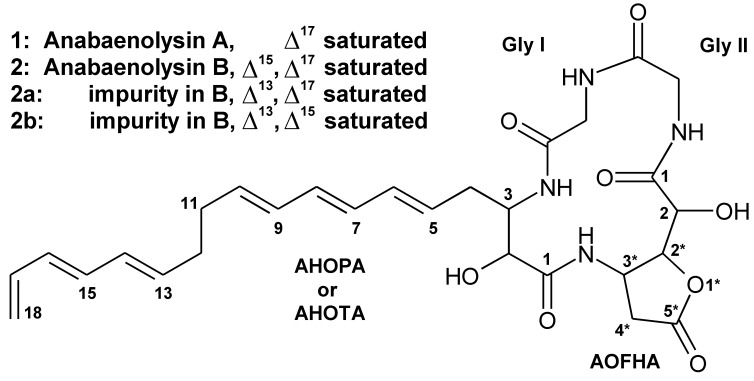
The structure of anabaenolysin A (1) and B (2)with the main (∼65%) and minor (∼5 and ∼30%) variants. AOFHA  = 2-(3-amino-5-oxotetrahydrofuran-2-yl)-2-hydroxyacetic acid. AHOPA  =  (5E,7E,9E,13E,15E)-3-amino-2-hydroxyoctadeca-5,7,9,13,15-pentaenoic acid. AHOTA  =  (5E,7E,9E,13E)-3-amino-2-hydroxyoctadeca-5,7,9,15-tetraenoic acid.

We screened about 30 other cyanobacterial strains (listed in Table 1 in ref [13]) of various genera for similar cytolytic activity and found significant presence of such activity in 5 isolates, all benthic *Anabaena* strains. The highest activity was detected in *Anabaena* strain XSPORK 27C. To know whether this cytolytic activity could originate from a new type of toxins the substances responsible for the activity were purified from *Anabaena* strains XPORK 15F and XSPORK 27C. HPLC chromatograms of the solid phase extracts are presented in Fig. 1E and F. The absorbance spectra of the purified cytolytic compounds (HPLC peaks containing the activity) from XPORK 15F and XSPORK 27C were similar between 250 and 300 nm, but differed at the region from 200 nm to 250 nm (inserts in Fig. 1E, F). This suggests that these cytolytic activities from strains XPORK 15F and XSPORK 27C were associated with two distinct, but probably related, molecules, and were termed anabaenolysin A (**1**) and B (**2**), respectively (Fig. 2).

**Table 1:** ^1^H, ^13^C and ^15^N NMR spectral data for anabaenolysin A and B in [D_6_]DMSO.

		Anabaenolysin A		Anabaenolysin B	
Substructure	C/H no	δC/δNa	δH, mult., J (Hz)b	δC	δHb
Gly I	CO	168.2		169.6	
	2	42.3	3.33, dd (3.7, 15.3)	43.5	3.35
	2′		3.80, dd (6.7, 15.3)		3.85
	NH	−268.5	7.58, dd (3.7, 6.7)		7.70
Gly II	CO	169.0		170.4	
	2	42.6	3.55	43.3	3.55
	2′		3.82, dd (6.9, 15.7)		3.88
	NH	−263.9	8.68, dd (4.7, 6.9)		8.77
AOFHA	CO	170.4		171.3	
	2-OH		5.84, d (5.2)		5.82
	2	70.4	4.20, dd (5.2)	71.6	4.26
	2^*^	84.9	4.54, dd	86.4	4.62
	3^*^	44.5	4.40, dddd (3.0, 3.0, 7.3, 9.9)	45.7	4.47
	3^*^-NH	−252.2	8.13, d (7.3)		8.27
	4^*^	35.4	2.24, dd (3.0, 17.8)	36.3	2.28
	4^*^,′		2.72, dd (9.9, 17.8)		2.77
	5^*^-CO	175.6		175.1	
AHOPA/AHOTA	CO	171.9		172.4	
	2	71.1	3.73, dd (2.1, 5,49)	72.3	3.79
	2-OH		5.57		
	3	51.1	3.93, m (2.1, 8.8)	52.7	3.99
	3-NH	−256.1	7.54, d (8.8)		7.60
	4	32.6	2.16	33.9	2.20
	4′		2.30		2.31
	5	129.9	5.55	131.1	5.58
	6	132.1	6.07	130.8	6.07
	7	130.5	6.07	131.9	6.07
	8	131.2	6.06	133.0	6.11
	9	130.5	6.03	130.2	6.07
	10	133.4	5.64, dt (14.0)	139.8	5.79
	11	31.6	2.10	33.1	2.05
	12	31.3	2.08	33.1	2.05
	13	130.9	5.51, m (6.4, 14)	131.1	5.58
	14	130.6	5.96	135.9	5.68
	15	129.0	5.94	33.1	2.05
	16	133.7	5.57	33.0	1.35
	17	24.5	1.98, dq (7,3)	23.0	1.27
	18	13.2	0.91, t (7.3)	14.2	0.87

Atom numbering is presented in Figure 2.

Numbering starts at the carboxyl carbon. AOFHA side chain numbers are marked with *.

a Relative to NO^3−^, ^b^ **δH** relative to TMS and *J* values of AHOPA methine protons 5…16 between 13…15 Hz measured from HSQC. AOFHA = 2-(3-amino-5-oxotetrahydrofuran-2-yl)-2-hydroxyacetic acid. AHOPA  =  (5E,7E,9E,13E,15E)-3-amino-2-hydroxyoctadeca-5,7,9,13,15-pentaenoic acid. AHOTA  =  (5E,7E,9E,13E)-3-amino-2-hydroxyoctadeca-5,7,9,15-tetraenoic acid.

### Anabaenolysin A (1) is a Novel Cyclic Lipopeptide with a Lactone Moiety and an Unsaturated C18 Hydrocarbon Chain

In the ion trap mass spectrometer **1** formed intense mono- (m/z 559 [M+H]^+^ and m/z 581 [M+Na]^+^), di- (m/z 1117 [2M+H]^+^ and m/z 1139 [2M+Na]^+^) and also trimeric (m/z 1697 [3M+Na]^+^) ions.

The ^1^H NMR spectrum of **1** in DMSO-*d_6_* revealed four amide (2°) type proton signals (two doublets and two double doublets) at δ 8.68, δ 8.13, δ 7.58 and δ 7.54 (Fig. S1). ^15^N HSQC connected these protons directly to 2°-amide type nitrogens (Fig. S2).

In ^1^H-^1^H TOCSY amide proton δ 7.58 (dd, NH^GlyI^) formed a spin system with protons δ 3.33 (H2^GlyI^) and δ 3.80 (H2’^GlyI^) and amide proton δ 8.68 (dd, NH^GlyII^) with protons δ 3.55 (H2^GlyII^) and δ 3.82 (H2’^GlyII^). The two pairs of 2,2′ protons were geminal based primarily on the coupling constants (Fig. S2, Table 1, Table S1) and on the correlations in the ^13^C HSQC (Fig. S3) spectrum although H2’^GlyI^ and H2’^GlyII^ correlations to the carbon signals δ 42.3 (C2^GlyI^) and δ 42.6 (C2^GlyII^) were not clearly resolved. ^13^C HMBC connected NH^GlyI^ and NH^GlyII^ to carbonyl carbons δ 168.2 (CO^GlyI^) and δ 169.0 (CO^GlyII^), respectively (Fig. S4). All this data showed that **1** contains two glycine residues.

In the AOFHA substructure of **1** ^1^H-^1^H TOCSY spectrum showed proton δ 8.13 (3*-NH) correlation to protons δ 2.24 (4*), δ 2.72 (4*′), δ 4.40 (3*) and δ 4.54 (2*) from which H2* further correlated to protons δ 4.20 (2) and δ 5.84 (2-OH) completing the spin system (Fig. S5). ^1^H-^1^H COSY connected 3*-NH to a methine proton 3* and it was further connected to H2* and geminal protons 4* and 4*′ (Fig. S6). Both geminal protons 4*,4*′ and proton 2* were connected to a carbonyl carbon δ 175.6 (5*-CO) (Fig. S4). Proton 2* was bonded to a carbon which shift value 84.9 ppm indicated direct bond to a heteroatom, preferably oxygen. Build up of a γ-lactone structure by bonding of the heteroatom to the aforementioned 5* carbonyl carbon explains the ^13^C HMBC correlation between proton 2* and 5*-CO (Fig. S4). ^1^H-^1^H COSY further connected 2* to a proton 2 that was bonded to a 70.4 ppm carbon atom (C2) indicating again direct bond to a heteroatom, preferably oxygen. As ^1^H-^1^H COSY linked proton 2 to a proton δ 5.84 (2-OH) which did not have a signal in ^13^C- or ^15^N HSQC it indicated a presence of a hydroxyl group. ^13^C HMBC correlation of protons 2 and 2-OH with carbonyl carbon δ 170.4 completed this substructure (Fig. S4). The above analysis led to a 2-(3-amino-5-oxytetrahydrofuran-2-yl)-2-hydroxyacetic acid (AOFHA) structure. Identical structure with matching carbon shifts has been described from another natural compound, amicoumacin C, which showed antibacterial activity against *Staphylococcus* strains [16].

In the AHOPAsubstructure ^1^H-^1^H COSY connected amide proton δ 7.54 (3-NH) to a methine proton δ 3.93 (H3), which was further linked to three protons δ 3.73 (H2), δ 2.16 (H4) and δ 2.30 (H4′) (Fig. S7). As H2 was bonded to a δ 71.1 carbon and coupled in ^1^H-^1^H COSY to a proton δ 5.57 (2-OH) which lacked a signal in ^13^C- or ^15^N HSQC, thus substructure probably contained a hydroxyl in C2. In ^13^C HMBC H2 had a correlation with carbonyl carbon δ 171.9 (Fig. S4),which formed the carboxyl terminal of this substructure. Hydrocarbon chain elongation from the methine proton H3 to H6 and from H9 to methyl protons H18 was clearly seen in ^1^H-^1^H COSY (Fig. S7 and S8). Chemical shifts of methine groups CH13, 14, 15 and 16 (Table 1) showed the presence of a conjugated diene structure. Together with signals δ 31.3 (C12) and δ 24.5 (C17), when compared to experimental and calculated signals from [17], the conjugated diene configuration was assigned as all-*trans* (EE). Examination of ^1^H-^1^H COSY together with ^13^C HSQC (Fig. S9) revealed that the crowded region from δ_H_ 6 to 6.1 contained the signals from H7 and 8 and crosspeaks almost fused to the diagonal. ^1^H-^1^H COSY and ^13^C HMBC connected CH7 and 8 together with CH 5, 6, 9 and 10 to a conjugated triene structure. Overlapping olefinic signals hindered the calculation of J values except in case of H10 that showed double triplet with a coupling constant of 14 Hz, which is typical for E double bond configuration. By comparing δ_CH_ signals of CH5…CH10 and δ_C_ signals of C4 and C11 with experimental and calculated signals from [17] and by taking into account the slight effect of electronegative nitrogen in β position to CH5, triene configuration was assigned as all-*trans* (EEE). Weak ^1^H NMR signals on the range from δ 5.0 to δ 5.4 and δ 6.2 to δ 6.5 could indicate variability in the double bond configuration. Altogether the aforementioned assignments led to a long chain polyunsaturated fatty acid structure, (5*E*,7*E*,9*E*,13*E*,15*E*)-3-amino-2-hydroxyoctadeca-5,7,9,13,15-pentaenoic acid (AHOPA). However, at this stage the stereochemistry of **1** at the five stereogenic centers (C-2, C-2*, C-3* in AOFHA; C-2 and C-3 in AHOPA) remained unassigned. COSY correlations of all subunits are summarized together with the HMBC correlations which link the subunits together in Fig. S10. Carbonyl region HMBC correlations important for subunit assembly are presented in Fig. S4 and all correlations are summarized in Table S1. Subunit assembly was possible without NOESY correlations.

To confirm the presence of glycine, a hydrolysate of **1** was analyzed with LC-MS after Marfey derivatization. From the UV (340 nm) chromatogram a peak corresponding to the Marfey derivative of glycine was identified (Fig. S11). Retention time, MS, MS^2^ and MS^3^ spectra were identical with reference FDAA-glycine. In addition, two masses of 175 Da and 193 Da were found, corresponding to the AOFHA with closed and open lactone ring structure (Fig. S11, Fig. 2).

The structure of **1** was studied with additional methods to ensure the structure assigned from NMR signals. The UV spectrum of **1** (Fig. 1E, insert) confirmed the presence of di-and tri- conjugated double bond structures in the molecule. Local λ_max_ at 228****nm (H_2_O/MeOH) is consistent with the well known λ_max_ at 227****nm of (2*E*, 4*E*)-hexa-2,4-diene. The UV spectrum of **1** contained also an absorption pattern characteristic for conjugated trienes. This pattern with λ_max_ at 262, 271 and 282 was practically identical with the pattern of (5*Z*, 7*E*, 9*E*, 14*Z*)-icosa-5, 7, 9, 14-tetranoic acid [18]. This double bond structure was in accordance with the AHOPA structure assigned from NMR studies.

The IR spectrum of **1** showed 1768 cm^−1^ signal coincident with C = O stretch of a 5 membered lactone structure, strong amide I bands at 1,657 cm^−1^ (C = O), 1,531 cm^−1^ (2° amide, C = O) and 995 cm^−1^ coincident with trans triene double bond.

The presence of two hydroxyl groups in the structure of **1**, one in AOFHA and another in AHOPA, was confirmed with 2-fluoro-1-methylpyridinium *p*-toluene sulfonate (FMP), which specifically reacts with the hydroxyl groups (Fig. S12). FMP reacted with **1** and formed two different mono-methylpyridinium (MP) derivatives (m/z 650 [M+H]^+^; R_t_ 32.9 min and 33.9 min; Fig. S12 C) and one di-MP derivative (m/z 371 [M+2H]^2+^; R_t_ 29.5 min; Fig. S12 D and the reaction scheme). These derivatives had the specific UV spectrum and strong absorption at 270 nm (Fig. S12 B) originating from the conjugated triene structure. Product ion spectra showed the loss of the MP group (Fig. S12 F–H). These results clearly confirmed the presence of two hydroxyls in **1**.

The presence and position of di- and tri-conjugated double bond structures in **1** was also studied with mass spectrometry. Compounds containing conjugated double bonds form covalent adduct when reacting with 4-methyl-1,2,4-triazoline-3,5-dione (MTAD) (Fig. S13). This MTAD adduct ion fragments on either side of the formed ring structure enabled the location of the double bonds. As expected **1** formed two different MTAD adducts (R_t_ 12.2 min and 12.9 min; Fig. S13 C and the reaction scheme), which in fragmentation formed product ions m/z 387 and m/z 544 from the adduct eluting at 12.3 min (Fig. S13 D) and m/z 413 (low intensity) and m/z 518 from the adduct eluting at 12.9 min (Fig. S13 E) giving additional evidence of the 5, 7, 9, 13, 15-pentaene structure of **1**.

High resolution MS m/z 559.27600 [M+H]^+^ (calculated for C_28_H_39_O_8_N_4_ −0.41****ppm error) was consistent with the structure assigned from the NMR, UV, IR and amino acid analysis data.

**Figure 3:**
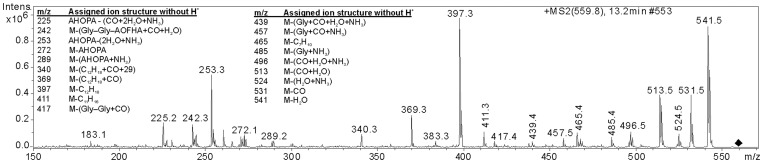
Product ion spectra of the protonated 1 and MS^3^ and assignments of the main product ions.

Fragmentation of protonated **1** was studied by using low accuracy MS^2−4^ (ion trap, Fig. S14 A) and high accuracy (LTQ orbitrap) spectra together with the MS^2^ spectrum from the ^15^N-labeled **1** (Fig. S14 B). All the most abundant ions could be assigned according to the presented structure of **1** with an average ion mass error less than 1 ppm (Figure 3, Table S2). Peak patterns 14 mass units apart from each other in MS^3^ spectrum from the ion m/z 253 aptly show the unsaturated hydrocarbon chain structure of the ion and its parent amino acid AHOPA (Fig. S14 C).

In conclusion, anabaenolysin A is a novel cyclic lipopeptide with the structure glycine^1^, glycine^2^, 2-(3-amino-5-oxytetrahydrofuran-2-yl)-2-hydroxyacetic acid^3,^ (5*E*,7*E*,9*E*,13*E*,15*E*)-3-amino-2-hydroxyoctadeca-5,7,9,13,15-pentaenoic acid^4^.

### Structure of Anabaenolysin B (2)

The structure of **2** (Fig. 2), as based on NMR analysis (Table 1, Table S3), was identical to that of **1** with the exception that AHOPA was replaced with (5*E*, 7*E*, 9*E*, 13*E*)-3amino-2-hydroxyoctadeca-5, 7, 9, 15-tetraenoic acid (AHOTA). The mono- and trienic AHOTA has one double bond less (15*E*) than the di- and trienic AHOPA. That structural difference is supported by the UV spectrum of **2**, which lacks the λ_max_ at 228****nm (Fig. 1F, insert), typical for dienes. Without the absorption at 228 nm there is a λ_min_ at 222 nm. **2** and cyclic lipotripeptide sclerotiotide A with a triene structure in the side chain share common structural features and also show similar absorption behavior near the typical peptide bond absorption area at 200–220 nm [19]. Impurities **2a** and **2b** in the purified **2** were revealed from the NMR data. In **2a** and **2b** AHOTA double bond structure was different as described in Fig. 2. Anabaenolysin B thus has the structure glycine^1^, glycine^2^, 2-(3-amino-5-oxytetrahydrofuran-2-yl)-2-hydroxyacetic acid^3^, (5*E*, 7*E*, 9*E*, 13*E*)-3amino-2-hydroxyoctadeca-5, 7, 9, 15-tetraenoic acid^4^.

### Evidence for Variants of Anabaenolysins

Aided by the annotated product ion spectra of **1** and to some extent the presence of the triene specific UV absorption pattern, we found evidence for the presence of 10 anabaenolysin variants in the *Anabaena* strain XPORK 15F (Table 2 and S4). **1** itself accounted for about 80% of the total anabaenolysin amount, as judged by the intensity of the [MH]^+^ signals (Table 2). Only anabaenolysin variants with 3 or more double bonds were detected so triene structure was present in all variants. The variants differed with respect to carbon chain length and the degree of unsaturation of the hydroxyamino-fatty acid (AHOPA in **1**). In variant series **1**, 3, 6 and 9 the chain length increased from C_16_ to C_19_, respectively, as the most intense fragment ion representing the fatty acid moiety of the anabaenolysins (m/z 253 in **1**, Table S2) increased in mass steps of 14 Da (Table S4). Another series is formed from variants **1**, **2**, 7 and 10 whose double bond number decreases successively from 6 to 3. This is seen from the fragment ion (m/z 253 in **1**, Table S2), which mass increased in steps of 2 Da (Table S4). Variants 4 and 5 may belong to the same series since their [MH]^+^ and retention times match exactly the mass versus retention time relationship of variants **1**, **2**, 7 and 10 (Table 2). Product ion spectra of variants 4 and 5 did not contain the ions characterising the carbon chain structure possibly because of the altered fragmentation at high unsaturation. Variant 8 was distinct by having an increased mass of 14 Da (potential CH_2_ unit) in the ion M-FA (m/z 272; M-AHOPA in **1**; Table S4).

**Table 2:** Anabaenolysin variants found in the *Anabaena* strain XPORK 15F.

Variant	R_t_ (min)	MH^+^ (m/z)	RA (%) ^a^	n in C_n_-FA	n of DB’s ^b^
3	21.7	531	2	16 •	5
4	21.7	553	1	18	8 ▪
5	22.4	555	1	18	7 ▪
6	22.5	545	1	17 •	5
7	22.9	557	2	18	6 ▪
8	23.3	573	2	18	5
1, Abl A	23.5	559	78	18 •	5 ▪
2, Abl B	23.9	561	7	18	4 ▪
9	24.4	573	2	19 •	5
10	24.7	563	3	18	3 ▪

Variants form a series with 16 to 19 carbons (•) or 3 to 8 double bonds in the unsaturated hydroxyamino-fatty acid substructure of anabaenolysin (▪). ^a^Relative abundancies (RA) calculated from the MH^+^ signals, ^b^Number of double bonds in FA, FA  =  unsaturated hydroxyamino−fatty acid, Abl = anabaenolysin.

The maximum yield of purified anabaenolysins were 3.3 mg of **1** and 2 mg of **2** per 8 g freeze-dried biomass from the strains XPORK 15F and XSPORK 27C, respectively. Extracellular anabaenolysins were not analysed during this study but later measurements performed with a new anabaenolysin variant (change in one of the glycines) found from another benthic *Anabaena* strain showed that only few percentage of the anabaenolysin was extracellular. Maybe the reason was just cell lysis, not active transport. Such a high abundance of one compound in an organism suggests that the anabaenolysins are important molecules for the cyanobacteria. Non-cyclic lipopeptides from *Anabaena* species have been shown to inhibit growth of *Microcystis* cyanobacteria [10]. Large cyclic lipopeptides from bacteria have reported to induce plant defence mechanisms [20]. Small cyclic lipotetrapeptides chlamydocins, trapoxins, HC-Toxin, Cyl−1 and −2, WF-3161, apicidins, azumamides and microsporins have been described from fungi and their phytotoxic bioactivities are best explained by the histone deacetylase inhibition [21]. Cyclic fungal fusaristatin lipotetrapeptides with a C20 dienoic moiety showed inhibition against lung cancer cells [22]. Cyclic fungal tripeptides aspochracin and sclerotiotides with a conjugated trienoic C8 fatty acid side chain showed antifungal activity but no cytotoxicity against HL-60 cell line [19]. Anabaenolysins being cyclic lipotetrapeptides are after all structurally considerably distinct due to the long fatty acid conjugated polyenoic moiety and the extraordinary amino acid AOFHA. It seems that the overall structure of anabaenolysins is unique which as well may lead to a characteristic bioactivity profile. The benthic *Anabaena* strains producing the anabaenalysins were all collected from biofilms [13]. Many organisms living in biofilms produce compounds for chemical defence [23], and this could explain the abundance of these amphiphilic, lytic compounds in our strains. However, further studies remains to conclude why the benthic *Anabaena* strains produce such large quantities of anabaenolysins.

### Anabaenolysin A (1) and B (2) were Cytotoxic towards Several Mammalian Cell Types and Induced Hemolysis

**Figure 4:**
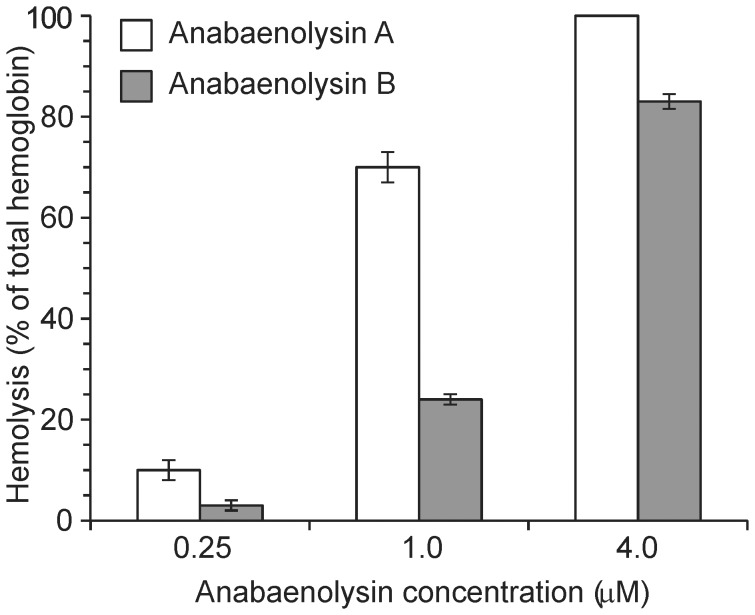
Hemolysis of isolated erythrocytes by different concentrations of anabaenolysin A and B.

We next tested isolated **1** and **2** for ability to induce cell death in different mammalian cell lines (Table 3). The LC_50_ values ranged from 4.4 to 14 µM for **1**, and 3.7 to 17 µM for **2**. Although no cell type stood out as particularly sensitive or resistant, we noted that suspension cells were more sensitive towards the anabaenolysins than adherent cells. This could be because a larger proportion of the cell membrane was available for attack by the anabaenolysins in suspension cells, and suggests that the anabaenolysins attack fundamental components in the outer cell membrane. The photomicrographs in Figure 1 B and C suggested that the anabaenolysins permeabilized the outer cell membrane. We therefore tested if they could induce hemolysis in isolated erythrocytes. **1** was more potent than **2**, inducing some hemolysis at 0.25 µM (Fig. 4). This was in line with the cytotoxicity data, where anabaenolysin A had higher potency towards most cell lines (Table 3).

**Table 3:** Concentrations of anabaenolysin A and B (in µM) inducing cell death in 50% of the cells a.

Cell line/Cells		Disease/normal cells	Anabaenolysin A	Anabaenolysin B
Jurkat	s ^b^	T-cell lymphoma	4.5±0.73	9.0±0.58
HL-60	s	AML	5.5±0.41	6.3±0.29
NB4	s	AML	6.5±0.20	3.7±0.12
IPC-81	s	AML	10±0.68	12±0.26
Ewing	a	Ewing Sarcoma	5.0±0.63	6.1±0.37
SHSY-5Y	a	Neuroblastoma	11±0.35	13±0.73
HeLa	a	Adenocarcinoma	14±1.70	17.0±0.79
U87	a	Glioblastoma/astrocytoma	10±1.20	8.5±0.26
NRK	a	Normal epithelial cells	11±0.31	11±0.29
Primary hepatocytes	s	Primary normal cells	4.4±0.22	11±3.20

a Values are mean of 3–5 experiments ± SEM, ^b^s denotes suspension and a adherent during the experiment.

## Materials and Methods

### Materials

Hoechst 33342, acetonitrile (Rathburn), 4-methyl-1,2,4-triazoline-3,5-dione (MTAD), glycine, and 2-fluoro-1-methylpyridinium *p*-toulene sulfonate (FMP) were from Sigma-Aldrich (St. Louis, USA). Dulbeccos Modified Eagle Medium (DMEM), horse serum and fetal bovine serum were supplied by EuroClone, Milano, Italy. RPMI 1640 Medium (Gibco) was from Invitrogen, Carlsbad, USA. All other chemicals were from VWR, West Chester, USA.

### Isolation and Cultivation of Cyanobacteria

Cyanobacteria strains XPORK 15F and XPORK 27C were isolated from the Baltic Sea coast at Porkkala, Finland (Gulf of Finland). The strains were identified as *Anabaena* sp. based on morphology according to [24] and sequencing of the 16S rRNA [25]. Mass cultivation, biomass collection and freeze-drying were as described in [13]. The planktonic microcystin producing *Anabaena* strain 318 was collected near station 39A close to Helsinki, Finland.

^15^N-labeled **1** was obtained as described by [26], except that ^15^N-urea (98+ % ^15^N, ISOTEC, USA) was used as nitrogen source and nitrogen-free argon (with 20.9% O_2_ and 0.45% CO_2_; quality 5.7; AGA Gas Ab, Sweden) was bubbled into the medium to prevent nitrogen fixation from air by the *Anabaena*. To maximize the degree of ^15^N-label in **1** *Anabaena* was successively cultivated three times by inoculating new culture with the biomass from the preceding culture. The cells from the third batch were used in LC-MS analysis. Final ^15^N content in **1** was estimated to be 75%.

### Purification of a Cytolytic Activity

Bioguided isolation of **1** and **2**: Freeze-dried biomass (1.2 g) was added to 60 ml of ice-cold methanol, ultrasonicated for 10 min. Thereafter 40 ml of methanol was added and the suspension was extracted for two hours on ice before centrifugation at 16000×g for 15 min at 4°C. Deionized water was added to supernatant to reach an 80/20 v/v methanol/water solution, and the solution was loaded onto a reversed phase solid phase extraction (SPE) column (Sep-Pak®Vac 35cc 10****g C18 cartridges, Waters, Milford, USA) and the cytolytic activity was eluted with 100 ml 80/20 (v/v) methanol/water. The bioactive flow through and wash fractions were combined, evaporated and dissolved in 60/40 (v/v) methanol/water before HPLC chromatography (Hitachi LaChrom; VWR, West Chester, USA with D-7000 interface, L-7150 pump, L-7400 UV detector and L-7612 degasser) using a reversed phase column (Kromasil®KR-100-10-C18, 250****mm×10mm, EKA, Bohus, Sweden), flow rate of 2.5 ml/min and mobile phase of 45/55 (v/v) water/acetonitrile.

Isolation of **1** and **2** for chemical identification: Freeze-dried biomass (2 g) was added to 30 ml of methanol and homogenised 3×20 s pulses (Silentcrusher M, Heidolph Instruments, Schwabach, Germany) with cooling on ice. Alternatively 0.2 g of biomass was put into 2 ml plastic tubes together with 0.55 g of 0.5 mm glass beads (Scientific Industries Inc.) and 1 ml of MeOH. The biomass was homogenized with FastPrep (Savant Instruments) 3×20 s in RT (5.0 m/s, with cooling in ice between the rounds). Extract volume corresponding to 1 g biomass was loaded onto a conditioned solid phase extraction cartridge (Strata C18-E; 50 µM, 70 Å, 5 g/ml, Phenomenex, Torrance, CA) and eluted with acetonitrile:methanol (1∶1). Eluent containing the lytic activity were first concentrated, then diluted with equal volume of water and finally separated by HPLC on a Luna C18 column (250×10****mm, Phenomenex, Torrance, CA) with a Zorbax C8 guard column (Agilent, Palo Alto, USA), run in isocratic mode with acetonitrile:water [45∶55 (v:v)] at a flow rate of 5 ml min^−1^ at 30°C. The fractions with the cytolytic activity were diluted with equal volume of water and concentrated on a RP-SPE cartridge conditioned with acetonitrile/water [15∶85 (v/v)] and then eluted with 10 ml 100% methanol. Effluent was evaporated, then dissolved in dimethylsulphoxide (DMSO) and re-injected into the HPLC. The collected fractions containing isolated anabaenolysins were pooled and evaporated. Anabaenolysins from the bioguided isolation was used to confirm that identical compounds were purified with this procedure.

### Chemical Analysis

LC-MS was performed on an Aglient 1100 Series LC/MSD TRAP System HPLC (Aglient Technologies, Palo Alto, USA) with XCT Plus model ion trap mass detector. Mass spectra were acquired using electrospray ionization in positive mode.

NMR spectra were recorded in [D_6_]DMSO at room temperature using a Varian Unity Inova 500 MHz NMR spectrometer for **2** and 600 MHz NMR spectrometer for **1**.

IR spectra (350–4000 cm^−1^) were recorded on a Bruker Vertex 70 FTIR spectrometer (Bruker Optics, Karlsruhe, Germany) equipped with a microplate HTS-XT accessory unit. Dissolved peptide was placed on a silicon plate, evaporated and the transmission spectrum was recorded.

**1** was hydrolyzed with 6N HCl for 19****hours at 110°C and after vacuum evaporation of solvent, residue was dissolved in water. Marfey derivatization (with FDAA, Pierce) of amino acids was performed as described by [9]. Marfey derivative of commercial glycine was prepared as a reference.

**1** was derivatized according to [27] with 4-methyl-1,2,4-triazoline-3,5-dione (MTAD, Sigma-Aldrich), which reacts with conjugated double bonds. The derivative was dissolved in methanol. In addition, **1** was derivatized according to [28] with 2-fluoro-1-methylpyridinium *p*-toulene sulfonate (FMP, Sigma-Aldrich), which reacts with alcohol groups. Both derivatives were analyzed by LC-MS as described above.

### Isolation of Primary Rat Hepatocytes, and Maintenance of Cell Lines

Primary rat hepatocytes were isolated from male Wistar rats (80–150****g) using *in vitro* collagenase perfusion [29], as previously described [30]. The rats had access to standard feed and water ad libitum, and the experiments were approved by the Norwegian Animal Research Authority and conducted according to the European Convention for the Protection of Vertebrates Used for Scientific Purposes. IPC-81 rat acute myeloid leukemia cells [31] were cultured in Dulbecco’s modified Eagle’s medium with 10% (v/v) heat inactivated horse serum. Human NB4 promyelocytic leukaemia cells [32] and human HL-60 leukemia cells (ATCC: CCL-240) were cultured in RPMI 1640 medium with 10% (v/v) heat inactivated fetal bovine serum. All the adherent cells (Table 3) (NRK, ATCC: CRL-6509, SHSY-5Y, ATCC: CRL-2266, HeLa, ATCC: CCL-2, U87, ATCC: HTB-14) were cultured in Dulbecco’s modified Eagle’s medium with 10% (v/v) heat inactivated fetal bovine serum, except from Ewing cells (gift from Francoise Besancon, INSERM, Hospital, St. Louis, Paris, France), which were cultured in RPMI 1640 medium with 10% (v/v) heat inactivated fetal bovine serum. When the cells had reached 75% confluence, they were detached by mild trypsin treatment (0.33 mg/ml trypsin for 5 min at 37°C), washed and reseeded in fresh medium to 25% confluence. All cell lines were incubated at 37°C in a humid atmosphere of 5% CO_2_.

### Determination of Cytolytic Activity and Cell Death of Nucleated Cells

Hepatocytes to be assessed morphologically for cell death were handled as described in [13]. The membrane integrity of non-fixed hepatocytes was assessed by exclusion of the dye trypan blue. All suspension cell lines were centrifuged at 160×g for 4 min, resuspended in fresh medium, and 2×10^4^ ****cells seeded in 0.1 ml in a 96-well tissue culture plate. The adherent cells were detached by mild trypsin treatment and seeded (5000****cells/well of Ewing, SHSY-5Y, U87, U373, and 2500****cells/well for HeLa and NRK) in a 96-well tissue culture plate the day before the experiments and the medium was renewed at the day of the experiment. After incubation with potential death inducers the cells were fixed in 2% buffered formaldehyde (pH 7.4) with 0.005 mg/ml of the DNA-dye Hoechst 33342. The surface morphology was evaluated by phase-, and differential interference- microscopy, and the chromatin distribution by fluorescent microscopy.

## Supporting Information

Figure S1 **^1^H NMR spectra of anabaenolysin A.** A) low field range, B) range showing methine signals and C) whole spectrum.
(PDF)

Figure S2 ^1^H-^1^H TOCSY and ^15^N HSQC correlation of the amide protons.
(PDF)

Figure S3 **^13^C HSQC partial spectrum showing correlations without the RHC = CHR region.**
(PDF)

Figure S4 **^13^C-HMBC partial spectrum showing carbonyl region.**
(PDF)

Figure S5 **^1^H-^1^H TOCSY partial spectrum showing AOFHA correlations.**
(PDF)

Figure S6 **^1^H-^1^H COSY partial spectrum showing AOFHA correlations.**
(PDF)

Figure S7 **^1^H-^1^H COSY partial spectrum showing AHOPA correlations.**
(PDF)

Figure S8 **^1^H-^1^H COSY partial spectra showing AHOPA correlations at the regions from δ_H_ 1.9 to δ_H_ 2.4 and δ_H_ 5.4 to δ_H_ 6.1.**
(PDF)

Figure S9 **^1^H-^1^H COSY and ^13^C HSQC partial spectra showing AHOPA correlations at the regions from δ_H_ 5.45 to δ_H_ 6.10 and from δ_H_ 128.5 to δ_H_ 134.**
(PDF)

Figure S10 **Structure of Anabaenolysin A with COSY correlations (bold lines) linking germinal and vicinal protons together and ^13^C HMBC (arrows) correlations linking substructures to each other.**
(PDF)

Figure S11 **UV (340 nm) chromatogram of the Marfey derivatised amino acids from the hydrolysis of anabaenolysin A.** Glycine was identified by comparing the retention time and mass spectra (MS and MS^n^) to the FDAA-glycine prepared from commercial amino acid. 175****Da and 193 Da peaks correspond to the AOFHA structure with closed and open lactone ring structure.
(PDF)

Figure S12 **Derivatisation of anabaenolysin A with 2-fluoro-1-methylpyridinium (FMP) and LC-MS analysis of the reaction mixture.** A: Reference ion chromatogram of protonated anabaenolysin A (m/z 559, R_t_ 38.9 min). Chromatograms from the reaction mixture; B: UV (270 nm) chromatogram with several peaks showing coelution with anabaenolysin derivatives. C: Ion chromatogram of m/z 650 with two peaks (R_t_ 32.9 min and 33.9 min) representing single charged mono-MP derivatives of anabaenolysin A. D: Ion chromatogram of m/z 371 with a peak (R_t_ 29.5 min) representing double charged di-MP derivative of anabaenolysin A. E: Ion chromatogram of m/z 559 showing the absence of anabenolysin A in the reaction mixture. Product ion spectra from MP-anabaenolysin A derivatives. F: MS^2^ from the double charged di-MP derivative of anabaenolysin A. G: MS^2^ from the former eluting (R_t_ 32.9 min) single charged mono-MP derivative of anabaenolysin A. H: MS^2^ from the latter eluting (Rt 33.9 min) single charged mono-MP derivative of anabaenolysin A.
(PDF)

Figure S13 **Derivatisation of anabaenolysin A with 4-methyl-1, 2, 4-triazoline-3,5-dione (MTAD) and LC-MS analysis of the reaction mixture.** A: Reference chromatogram of protonated anabaenolysin A (m/z 559, R_t_ 20.2 min). Chromatograms from the reaction mixture; B: Ion chromatogram of m/z 559 showing the absence of anabaenolysin A in the reaction mixture. C: Ion chromatogram of m/z 785 with two peaks (R_t_ 12.2 min and 12.9 min) representing two different MTAD derivatives of anabaenolysin A. Product ion spectra from MTAD-anabaenolysin A derivatives; D: MS^2^ from the former eluting (R_t_ 12.2 min) MTAD derivative of anabaenolysin A showing characteristic ions m/z 387 and m/z 544. E: MS^2^ from the latter eluting (R_t_ 12.9 min) MTAD derivative of anabaenolysin A showing characteristic ions m/z 413 (low intensity) and m/z 518.
(PDF)

Figure S14 **Product ion spectra (MS^2^) from unlabeled (A) and ^15^N-labeled (B) anabaenolysin A and MS^3^ spectrum of ion m/z 253 (C).**
(PDF)

Table S1 **^1^H, ^13^C and ^15^N NMR spectral data for anabaenolysin A (1) in DMSO-d6.**
(PDF)

Table S2 **Product ion assignments from the fragmentation of the unlabeled and ^15^N-labeled protonated anabaenolysin A.**
(PDF)

Table S3 **^1^H, ^13^C and ^15^N NMR spectral data for anabaenolysin B (2) in DMSO-d6.**
(PDF)

Table S4 **Anabaenolysin variants found in the** ***Anabaena*** **strain XPORK 15F. Identification based on UV-spectra showing the triene specific absorption pattern, ion mass [MH]^+^, product ion spectra and retention time (R_t_).** Product ions presented characterise the differences in the structures.
(PDF)
