# Supplementary material for: Anabaenolysins, Novel Cytolytic Lipopeptides from Benthic Anabaena Cyanobacteria
Source: PLoS One. 2012 Jul 19;7(7):e41222. doi: 10.1371/journal.pone.0041222 (PMC3400675; doi:10.1371/journal.pone.0041222)
Supplement: Figure S1 — 1H NMR spectra of anabaenolysin A. A) low field range, B) range showing methine signals and C) whole spectrum. (PDF) [file pone.0041222.s001.pdf]

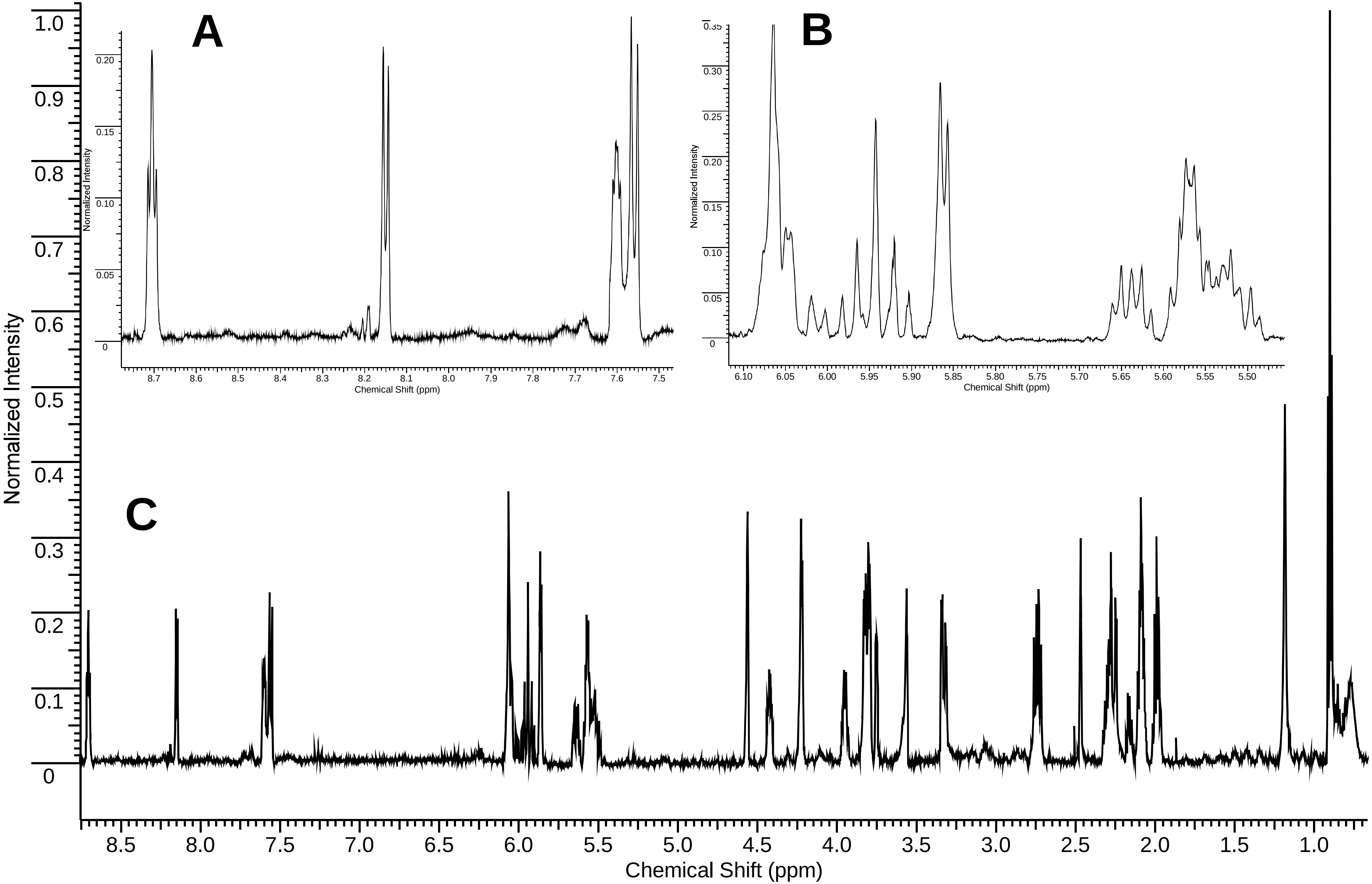


**Figure S1. $^{1}$H NMR spectra of 1 (Abl A). A) low field range, B) range showing methine signals and C) whole spectrum.**
