# Supplementary figures and images for: Anabaenolysins, Novel Cytolytic Lipopeptides from Benthic Anabaena Cyanobacteria
Source: PLoS One. 2012 Jul 19;7(7):e41222. doi: 10.1371/journal.pone.0041222 (PMC3400675; doi:10.1371/journal.pone.0041222)

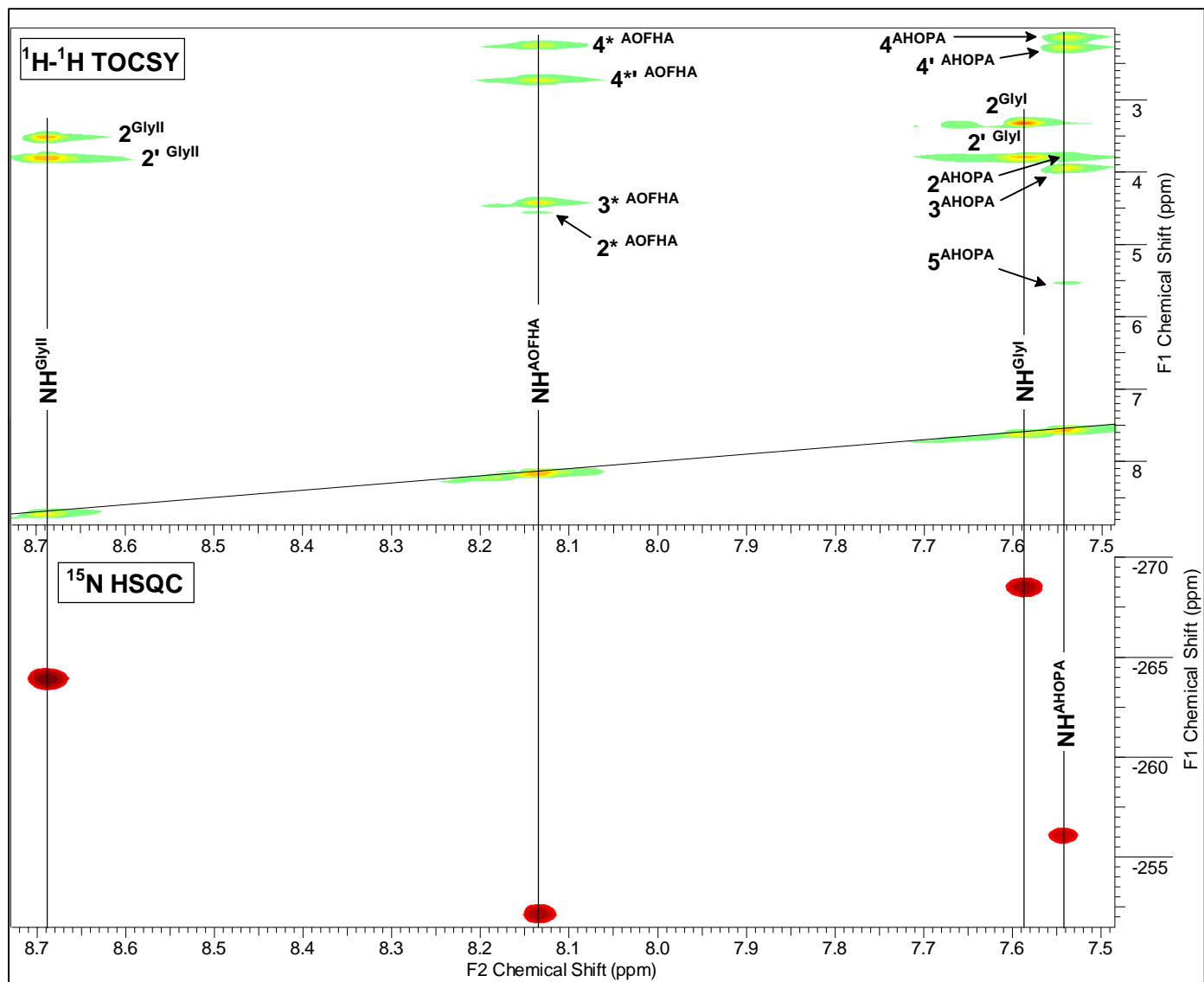

**Figure S2.**  $^1\text{H}$ - $^1\text{H}$  TOCSY and  $^{15}\text{N}$  HSQC correlation of the amide protons.

Supplement: Figure S2 — 1H-1H TOCSY and 15N HSQC correlation of the amide protons. (PDF) [file pone.0041222.s002.pdf]

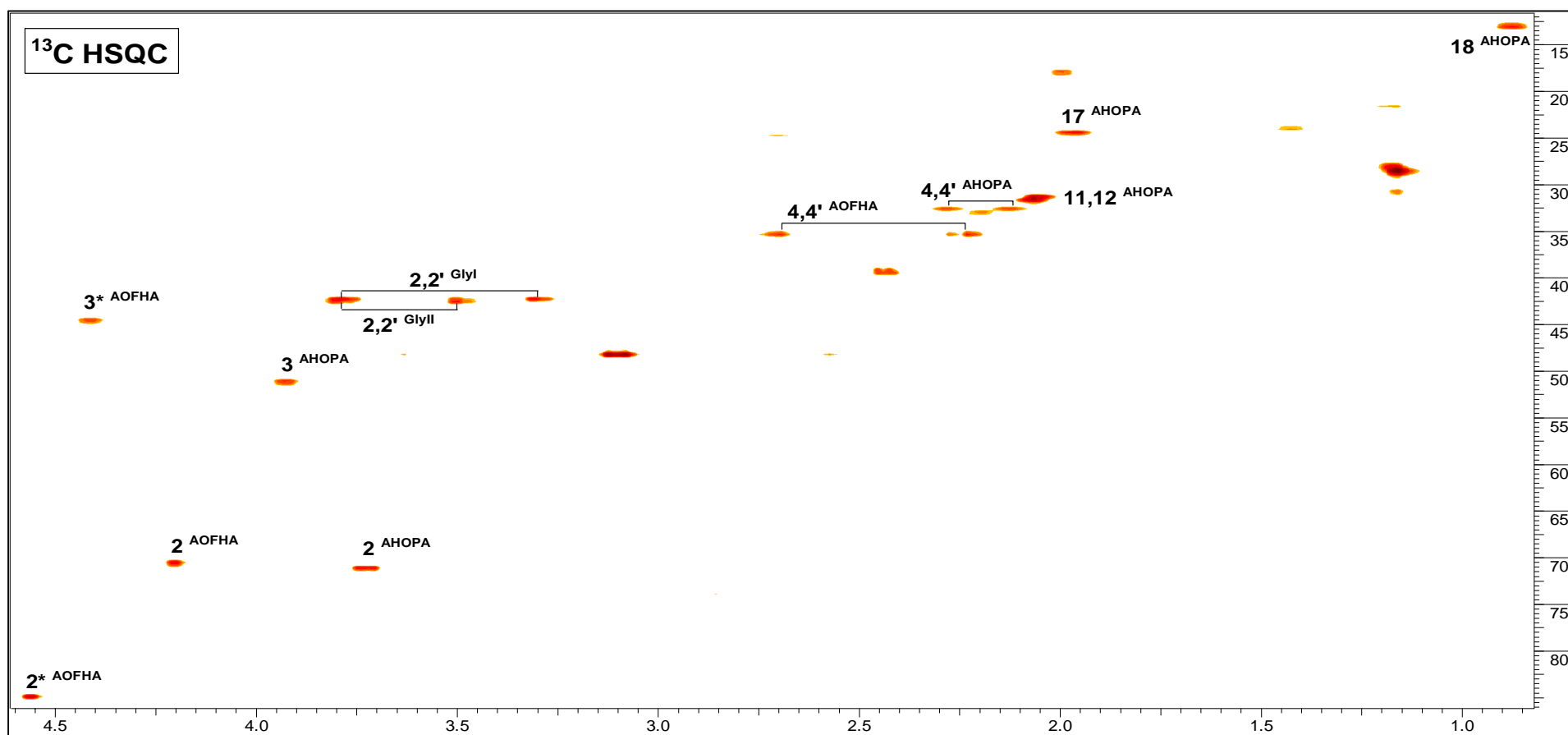

**Figure S3 HSQC.** <sup>13</sup>C-HSQC partial spectrum showing correlations without the RHC=CHR region.

Supplement: Figure S3 — 13C HSQC partial spectrum showing correlations without the RHC = CHR region. (PDF) [file pone.0041222.s003.pdf]

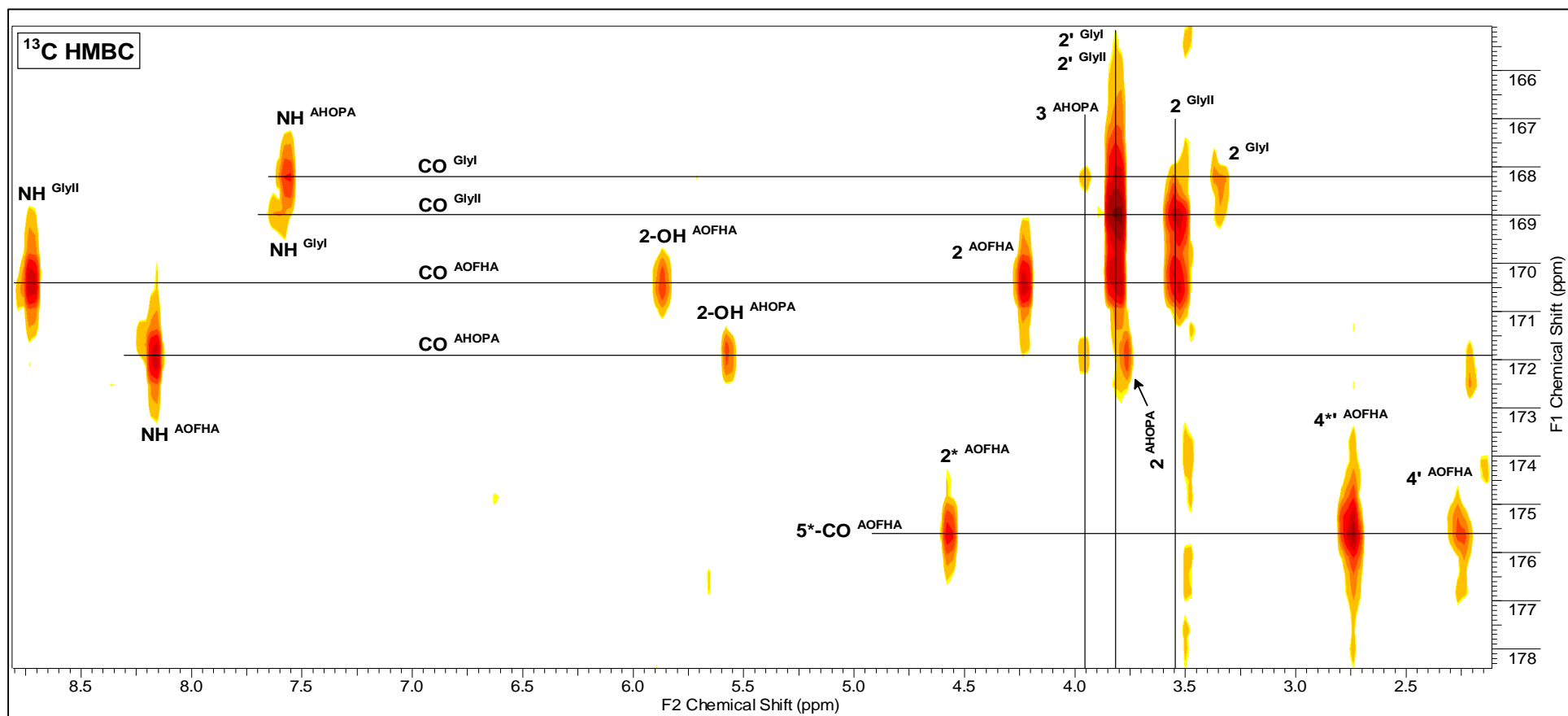

**Figure S4.**  $^{13}\text{C}$ -HMBC partial spectrum showing carbonyl region.

Supplement: Figure S4 — 13C-HMBC partial spectrum showing carbonyl region. (PDF) [file pone.0041222.s004.pdf]

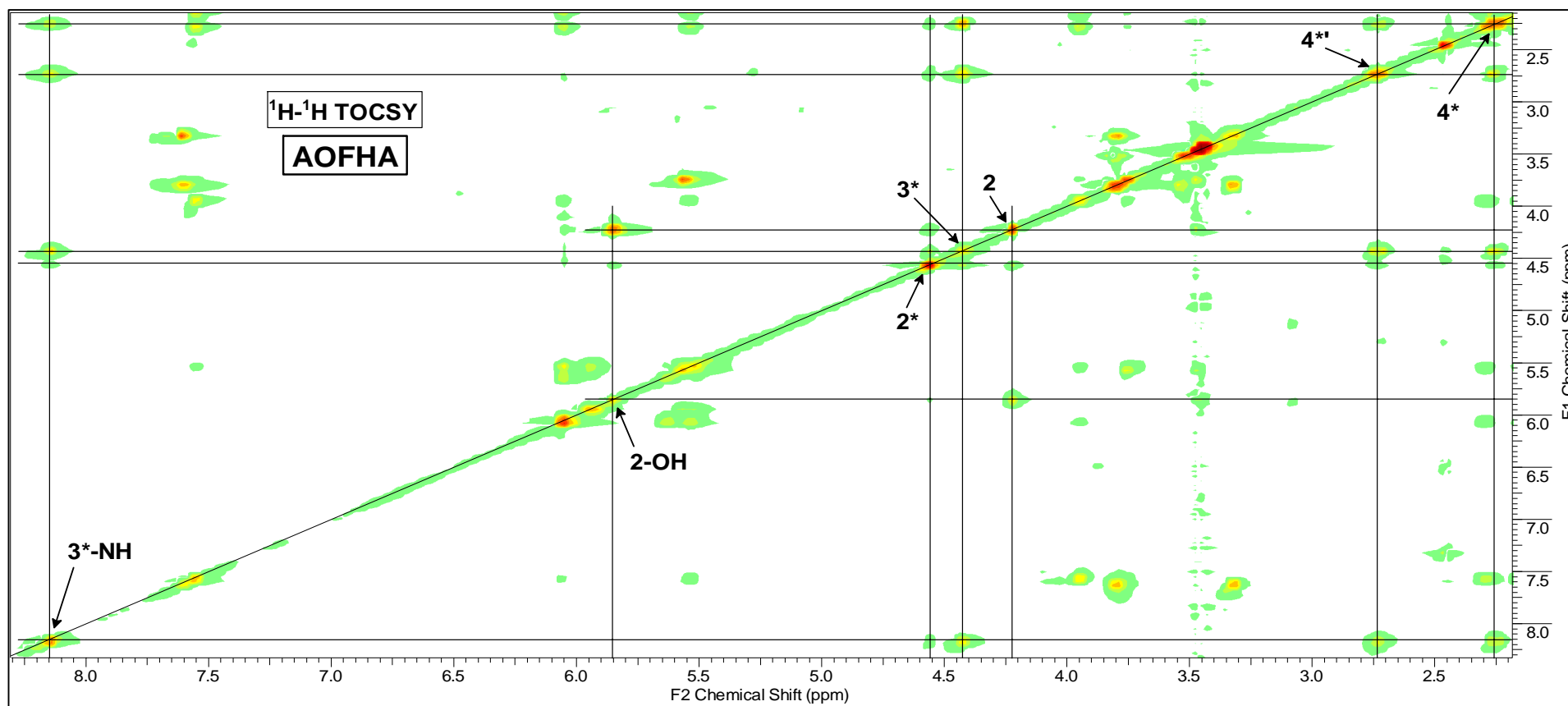

**Figure S5.**  $^1\text{H}$ - $^1\text{H}$  TOCSY partial spectrum showing AOFHA correlations.

Supplement: Figure S5 — 1H-1H TOCSY partial spectrum showing AOFHA correlations. (PDF) [file pone.0041222.s005.pdf]

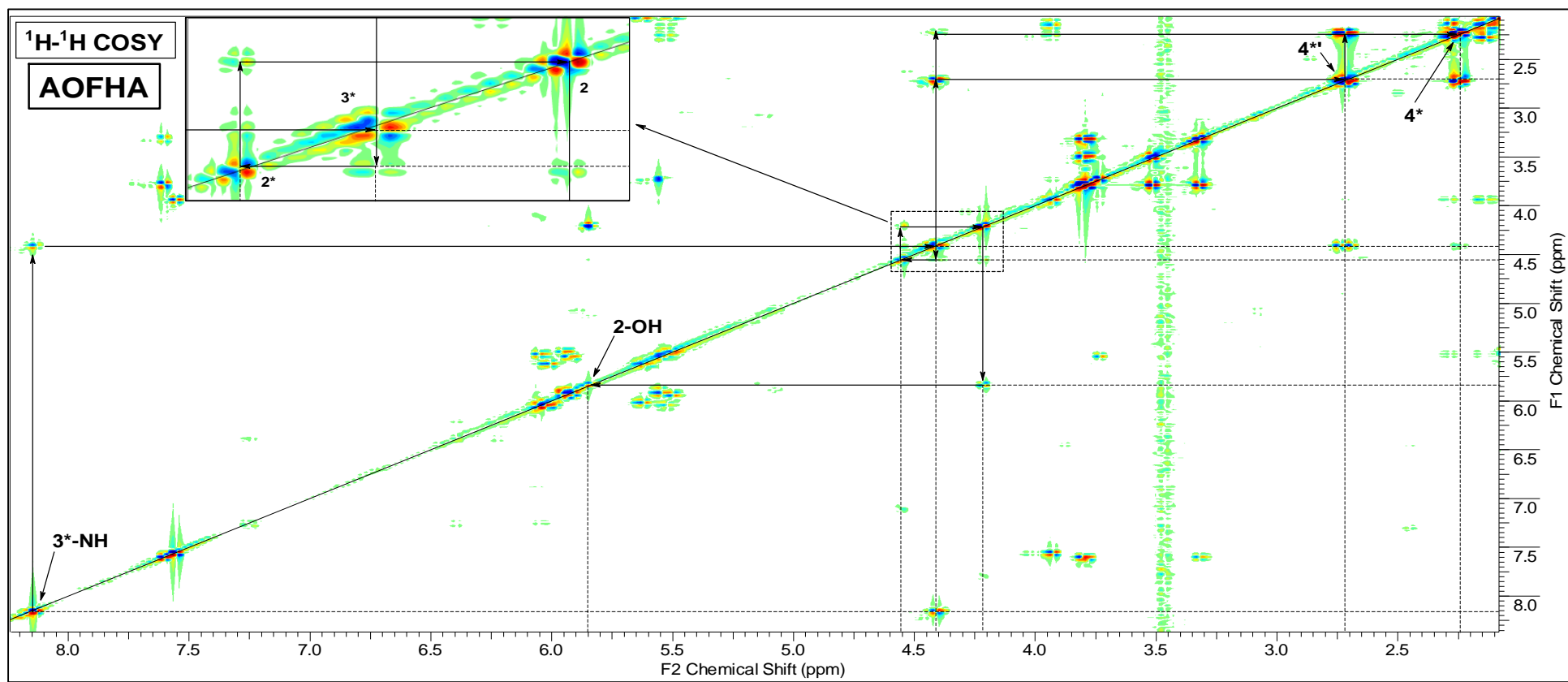

**Figure S6.**  $^1\text{H}$ - $^1\text{H}$  COSY partial spectrum showing AOFHA correlations.

Supplement: Figure S6 — 1H-1H COSY partial spectrum showing AOFHA correlations. (PDF) [file pone.0041222.s006.pdf]

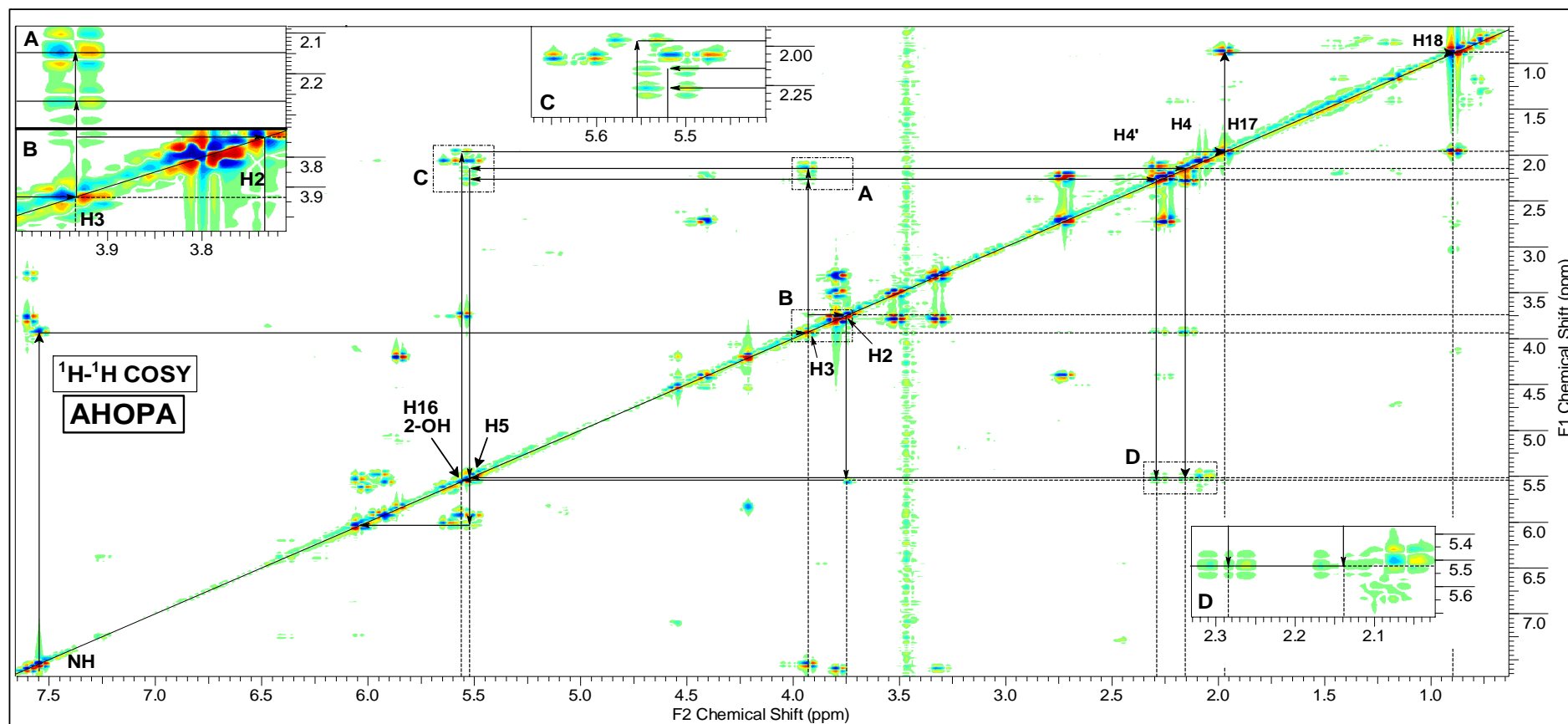

**Figure S7.**  $^1\text{H}$ - $^1\text{H}$  COSY partial spectrum showing AHOPA correlations.

Supplement: Figure S7 — 1H-1H COSY partial spectrum showing AHOPA correlations. (PDF) [file pone.0041222.s007.pdf]
