# Supplementary material for: Anabaenolysins, Novel Cytolytic Lipopeptides from Benthic Anabaena Cyanobacteria
Source: PLoS One. 2012 Jul 19;7(7):e41222. doi: 10.1371/journal.pone.0041222 (PMC3400675; doi:10.1371/journal.pone.0041222)
Supplement: Figure S8 — 1H-1H COSY partial spectra showing AHOPA correlations at the regions from δH 1.9 to δH 2.4 and δH 5.4 to δH 6.1. (PDF) [file pone.0041222.s008.pdf]

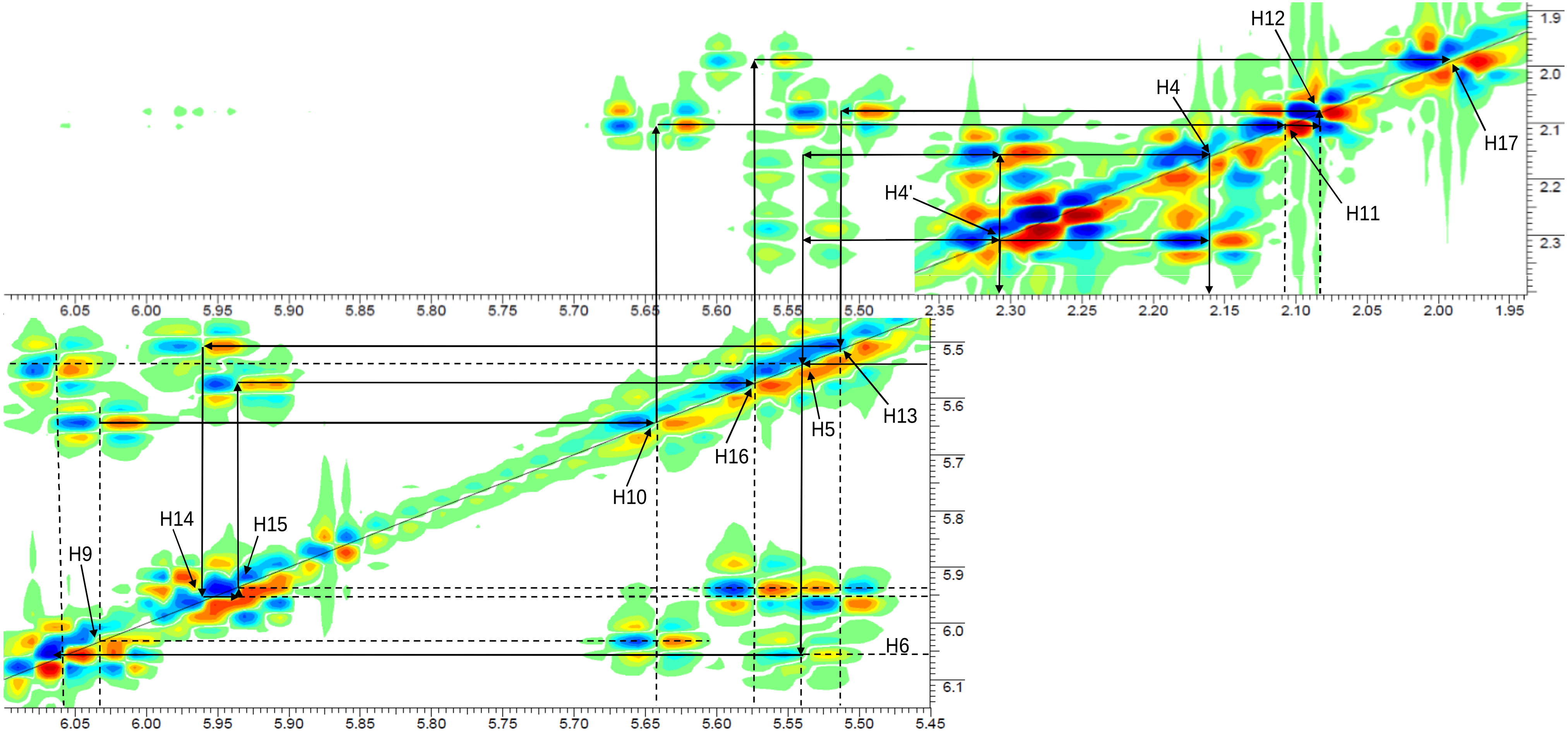

Figure S8. ^{1}H-^{1}H COSY partial spectra showing AHOPA correlations at the regions from δ_H 1.9 to δ_H 2.4 and δ_H 5.4 to δ_H 6.1.
