# Supplementary material for: Anabaenolysins, Novel Cytolytic Lipopeptides from Benthic Anabaena Cyanobacteria
Source: PLoS One. 2012 Jul 19;7(7):e41222. doi: 10.1371/journal.pone.0041222 (PMC3400675; doi:10.1371/journal.pone.0041222)
Supplement: Figure S9 — 1H-1H COSY and 13C HSQC partial spectra showing AHOPA correlations at the regions from δH 5.45 to δH 6.10 and from δH 128.5 to δH 134. (PDF) [file pone.0041222.s009.pdf]

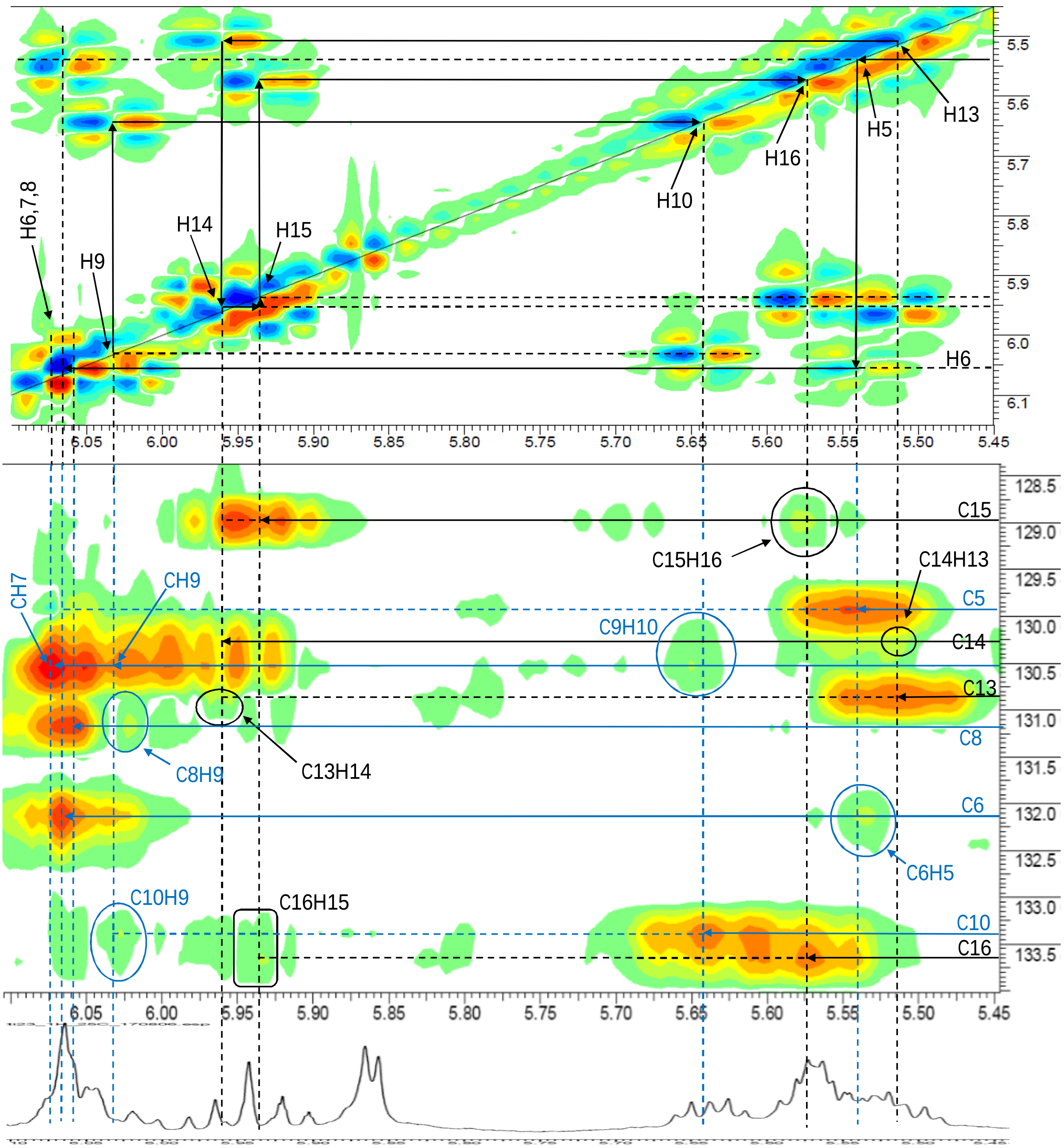

Figure S9. ^{1}H-^{1}H COSY and ^{13}C HSQC partial spectra showing AHOPA correlations at the regions from δH 5.45 to δH 6.10 and from δH 128.5 to δH 134.
