# Supplementary material for: Anabaenolysins, Novel Cytolytic Lipopeptides from Benthic Anabaena Cyanobacteria
Source: PLoS One. 2012 Jul 19;7(7):e41222. doi: 10.1371/journal.pone.0041222 (PMC3400675; doi:10.1371/journal.pone.0041222)
Supplement: Figure S11 — UV (340 nm) chromatogram of the Marfey derivatised amino acids from the hydrolysis of anabaenolysin A. Glycine was identified by comparing the retention time and mass spectra (MS and MSn) to the FDAA-glycine prepared from commercial amino acid. 175Da and 193 Da peaks correspond to the AOFHA structure with closed and open lactone ring structure. (PDF) [file pone.0041222.s011.pdf]

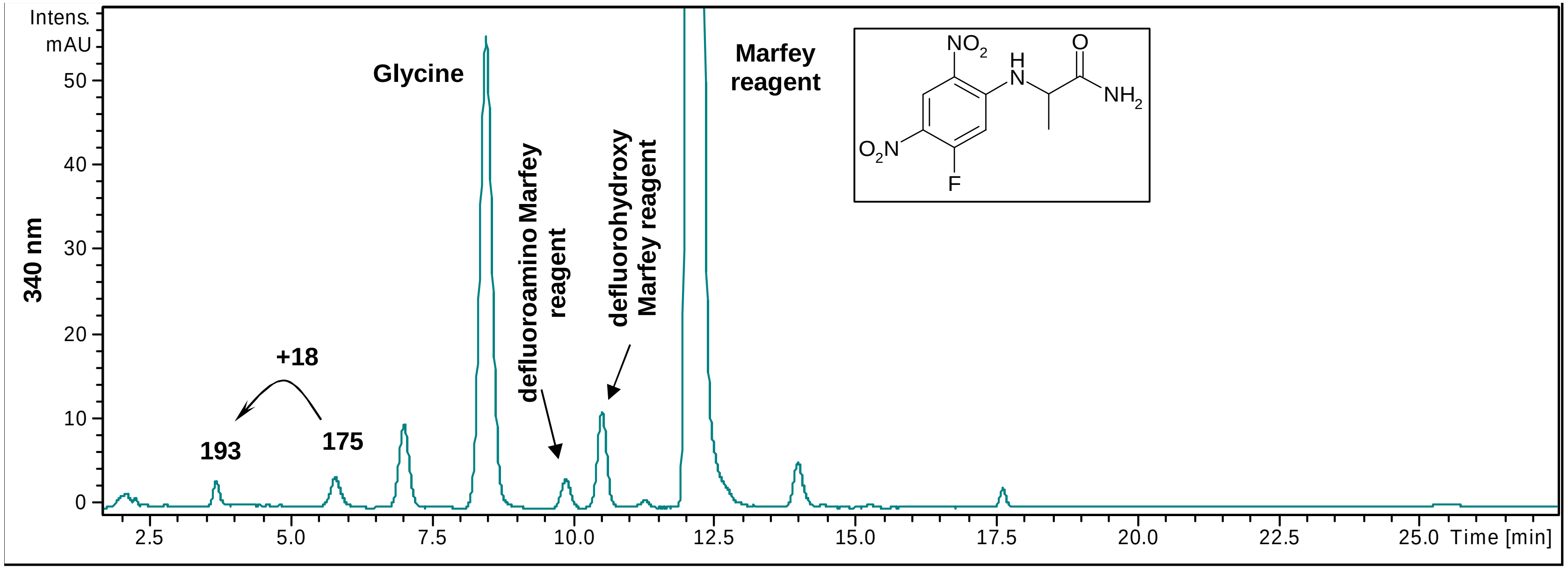

Figure S11. UV (340nm) chromatogram of the Marfey derivatised amino acids from the hydrolysis of anabaenolysin A. Glycine was identified by comparing the retention time and mass spectra (MS and MSn) to the FDAA-glycine prepared from commercial amino acid. 175Da and 193 Da peaks correspond to the AOFHA structure with closed and open lactone ring structure.
