# Supplementary material for: Anabaenolysins, Novel Cytolytic Lipopeptides from Benthic Anabaena Cyanobacteria
Source: PLoS One. 2012 Jul 19;7(7):e41222. doi: 10.1371/journal.pone.0041222 (PMC3400675; doi:10.1371/journal.pone.0041222)
Supplement: Figure S12 — Derivatisation of anabaenolysin A with 2-fluoro-1-methylpyridinium (FMP) and LC-MS analysis of the reaction mixture. A: Reference ion chromatogram of protonated anabaenolysin A (m/z 559, Rt 38.9 min). Chromatograms from the reaction mixture; B: UV (270 nm) chromatogram with several peaks showing coelution with anabaenolysin derivatives. C: Ion chromatogram of m/z 650 with two peaks (Rt 32.9 min and 33.9 min) representing single charged mono-MP derivatives of anabaenolysin A. D: Ion chromatogram of m/z 371 with a peak (Rt 29.5 min) representing double charged di-MP derivative of anabaenolysin A. E: Ion chromatogram of m/z 559 showing the absence of anabenolysin A in the reaction mixture. Product ion spectra from MP-anabaenolysin A derivatives. F: MS2 from the double charged di-MP derivative of anabaenolysin A. G: MS2 from the former eluting (Rt 32.9 min) single charged mono-MP derivative of anabaenolysin A. H: MS2 from the latter eluting (Rt 33.9 min) single charged mono-MP derivative of anabaenolysin A. (PDF) [file pone.0041222.s012.pdf]

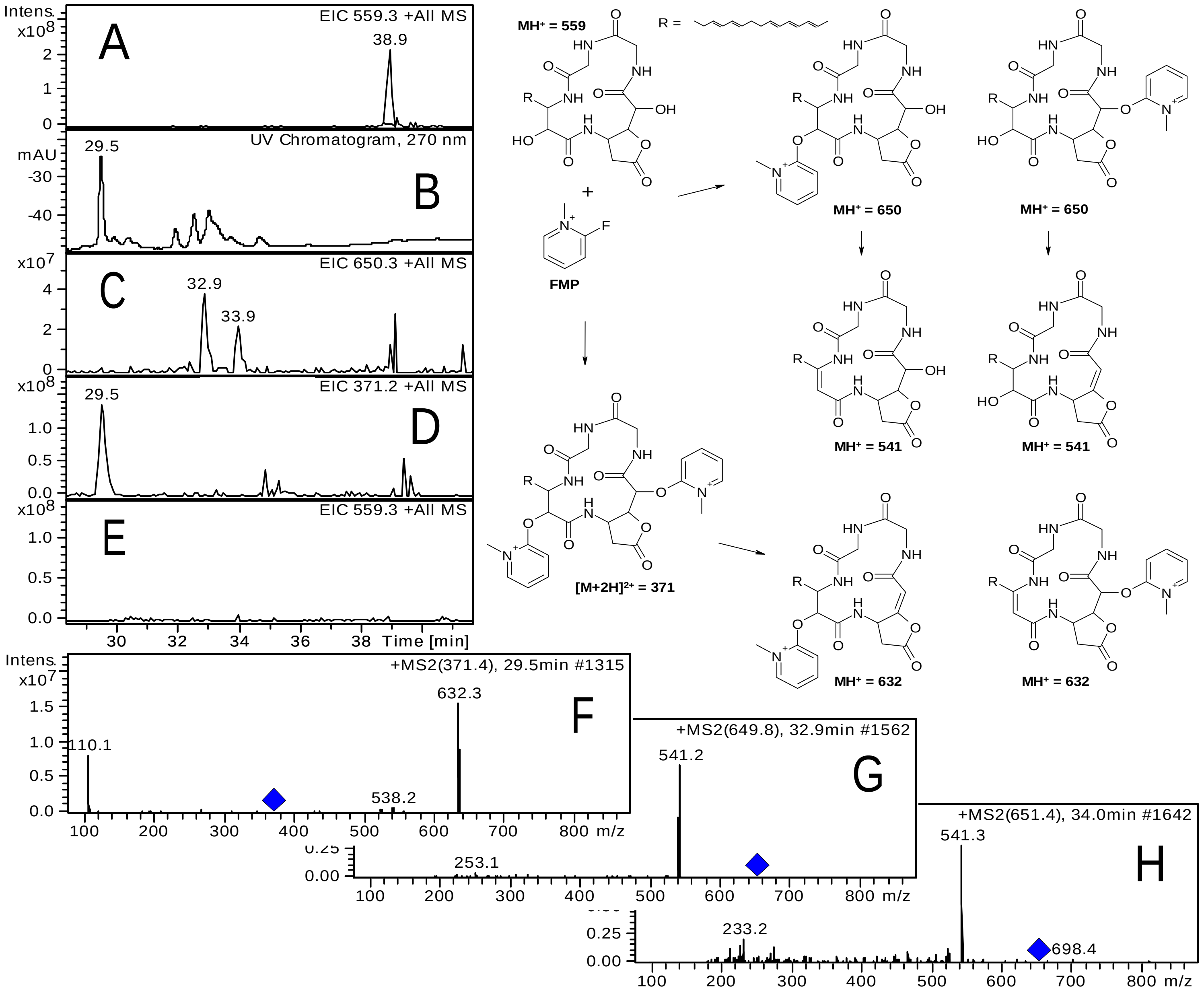

EIC 559.3 +All MS
38.9
UV Chromatogram, 270 nm
29.5
EIC 650.3 +All MS
32.9
33.9
EIC 371.2 +All MS
29.5
EIC 559.3 +All MS
Time [min]
+MS2(371.4), 29.5min #1315
632.3
110.1
538.2
+MS2(649.8), 32.9min #1562
541.2
253.1
+MS2(651.4), 34.0min #1642
541.3
233.2
698.4
MH+ = 559
R =
FMP
MH+ = 650
MH+ = 650
MH+ = 541
MH+ = 541
[M+2H]2+ = 371
MH+ = 632
MH+ = 632

Figure S12. Derivatisation of anabaenolysin A with 2-fluoro-1-methylpyridinium (FMP) and LC-MS analysis of the reaction mixture. A: Reference ion chromatogram of protonated anabaenolysin A (m/z 559, Rt 38.9 min). Chromatograms from the reaction mixture; B: UV (270 nm) chromatogram with several peaks showing coelution with anabaenolysin derivatives. C: Ion chromatogram of m/z 650 with two peaks (Rt 32.9 min and 33.9 min) representing single charged mono-MP derivatives of anabaenolysin A. D: Ion chromatogram of m/z 371 with a peak (Rt 29.5 min) representing double charged di-MP derivative of anabaenolysin A. E: Ion chromatogram of m/z 559 showing the absence of anabenolysin A in the reaction mixture. Product ion spectra from MP-anabaenolysin A derivatives. F: MS2 from the double charged di-MP derivative of anabaenolysin A. G: MS2 from the former eluting (Rt 32.9 min) single charged mono-MP derivative of anabaenolysin A. H: MS2 from the latter eluting (Rt 33.9 min) single charged mono-MP derivative of anabaenolysin A.
