# Supplementary material for: Anabaenolysins, Novel Cytolytic Lipopeptides from Benthic Anabaena Cyanobacteria
Source: PLoS One. 2012 Jul 19;7(7):e41222. doi: 10.1371/journal.pone.0041222 (PMC3400675; doi:10.1371/journal.pone.0041222)
Supplement: Figure S13 — Derivatisation of anabaenolysin A with 4-methyl-1, 2, 4-triazoline-3,5-dione (MTAD) and LC-MS analysis of the reaction mixture. A: Reference chromatogram of protonated anabaenolysin A (m/z 559, Rt 20.2 min). Chromatograms from the reaction mixture; B: Ion chromatogram of m/z 559 showing the absence of anabaenolysin A in the reaction mixture. C: Ion chromatogram of m/z 785 with two peaks (Rt 12.2 min and 12.9 min) representing two different MTAD derivatives of anabaenolysin A. Product ion spectra from MTAD-anabaenolysin A derivatives; D: MS2 from the former eluting (Rt 12.2 min) MTAD derivative of anabaenolysin A showing characteristic ions m/z 387 and m/z 544. E: MS2 from the latter eluting (Rt 12.9 min) MTAD derivative of anabaenolysin A showing characteristic ions m/z 413 (low intensity) and m/z 518. (PDF) [file pone.0041222.s013.pdf]

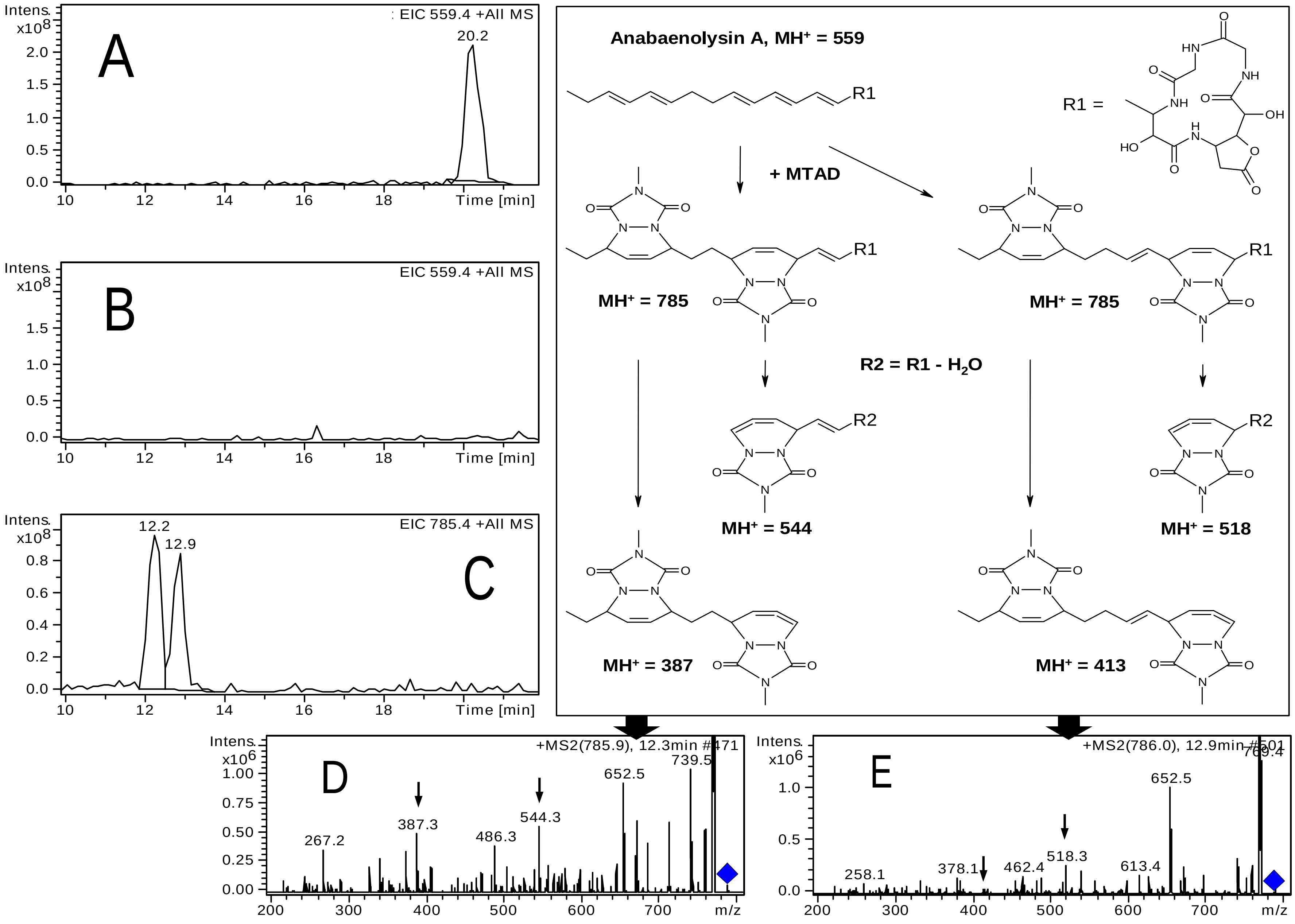

A
Intens. x10^8
: EIC 559.4 +All MS
20.2
Time [min]
B
EIC 559.4 +All MS
C
EIC 785.4 +All MS
12.2
12.9
Anabaenolysin A, MH+ = 559
R1
R1 =
+ MTAD
MH+ = 785
MH+ = 785
R2 = R1 - H2O
R2
MH+ = 544
MH+ = 518
MH+ = 387
MH+ = 413
D
+MS2(785.9), 12.3min #471
739.5
652.5
544.3
486.3
387.3
267.2
m/z
E
+MS2(786.0), 12.9min #501
769.4
652.5
518.3
613.4
462.4
378.1
258.1

Figure S13. Derivatisation of anabaenolysin A with 4-methyl-1, 2, 4-triazoline-3,5-dione (MTAD) and LC-MS analysis of the reaction mixture. A: Reference chromatogram of protonated anabaenolysin A (m/z 559, Rt 20.2 min). Chromatograms from the reaction mixture; B: Ion chromatogram of m/z 559 showing the absence of anabaenolysin A in the reaction mixture. C: Ion chromatogram of m/z 785 with two peaks (Rt 12.2 min and 12.9 min) representing two different MTAD derivatives of anabaenolysin A. Product ion spectra from MTAD-anabaenolysin A derivatives; D: MS2 from the former eluting (Rt 12.2 min) MTAD derivative of anabaenolysin A showing characteristic ions m/z 387 and m/z 544. E: MS2 from the latter eluting (Rt 12.9 min) MTAD derivative of anabaenolysin A showing characteristic ions m/z 413 (low intensity) and m/z 518.
