# Supplementary material for: Anabaenolysins, Novel Cytolytic Lipopeptides from Benthic Anabaena Cyanobacteria
Source: PLoS One. 2012 Jul 19;7(7):e41222. doi: 10.1371/journal.pone.0041222 (PMC3400675; doi:10.1371/journal.pone.0041222)
Supplement: Figure S14 — Product ion spectra (MS2) from unlabeled (A) and 15N-labeled (B) anabaenolysin A and MS3 spectrum of ion m/z 253 (C). (PDF) [file pone.0041222.s014.pdf]

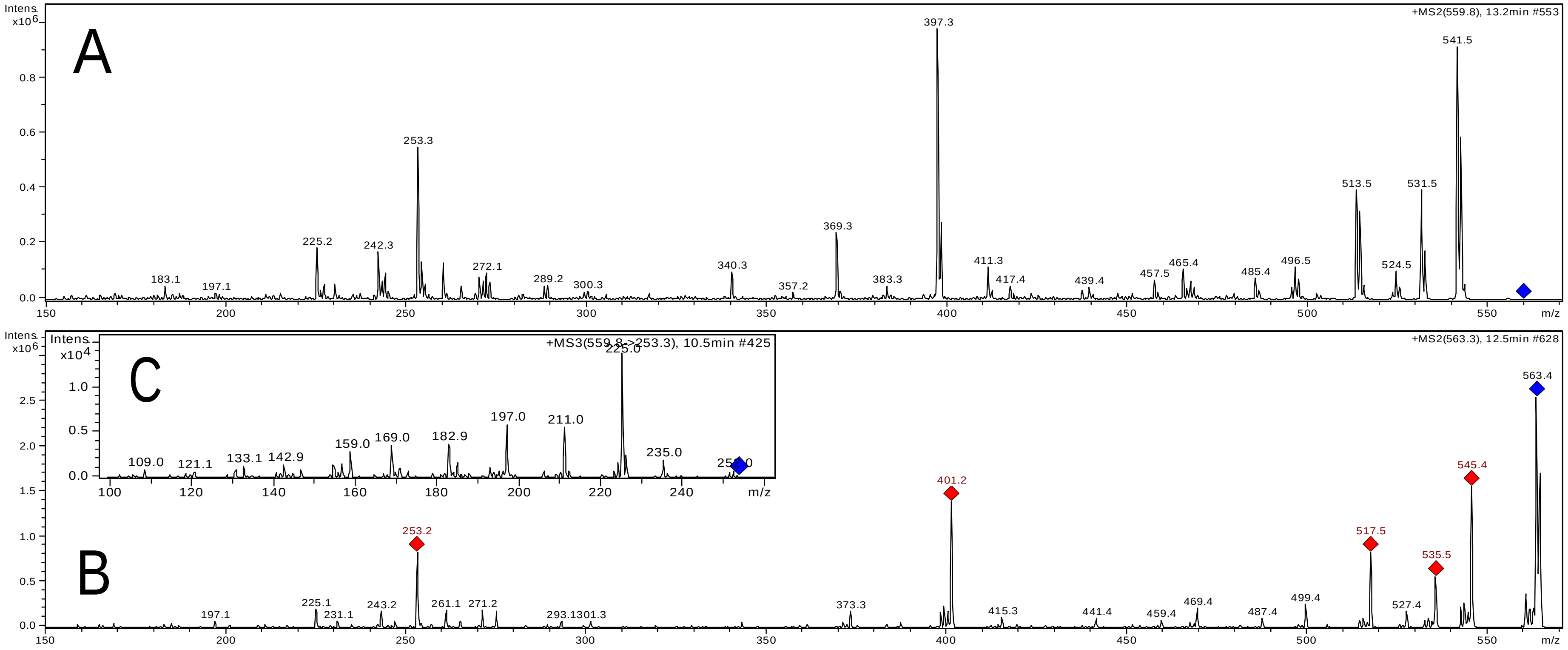

Figure S14. Product ion spectra (MS^2) from unlabeled (A) and ^{15}N-labeled (B) anabaenolysin A and MS^3 spectrum of ion m/z 253 (C).
