# Supplementary material for: Anabaenolysins, Novel Cytolytic Lipopeptides from Benthic Anabaena Cyanobacteria
Source: PLoS One. 2012 Jul 19;7(7):e41222. doi: 10.1371/journal.pone.0041222 (PMC3400675; doi:10.1371/journal.pone.0041222)
Supplement: Table S1 — 1H, 13C and 15N NMR spectral data for anabaenolysin A (1) in DMSO-d6. (PDF) [file pone.0041222.s015.pdf]

Table S1. ^{1}H, ^{13}C and ^{15}N NMR spectral data for anabaenolysin A (**1**) in DMSO-d6.

Substructure	C/H no	δC/δN[a]	δH, mult., *J* (Hz)[b]	COSY/TOCSY[c]	H2BC[d]	HMBC[d]	NOESY
Gly I	CO	168.2					
	2	42.3	3.33, dd (3.7, 15.3)	(2'), NH	2	1, GlyII-1	2', NH
	2'		3.8, dd (6.7, 15.3)	2, NH	2	1(overlap)	2, NH
	NH	-268.5	7.58, dd (3.7, 6.7)	2,2'	2	GlyII-1	2, 2', GlyII-2, NH, AOFHA-2*
Gly II	CO	169.0					
	2	42.6	3.55	(2'), NH	-	1, AOFHA-1	-
	2'		3.82, dd (6.9, 15.7)	2, NH	2	1, AOFHA-1	2, NH
	NH	-263.9	8.68, dd (4.7, 6.9)	2,2'	2	2, AOFHA-1	2,2', GlyI-NH, AOFHA-2, 2*
AOFHA	CO	170.4					
	2-OH		5.84, d (5.2)	2 / 2*	2	1, 2, 2*	2, 3*, 4*', GlyII-NH
	2	70.4	4.2, dd (1, 5.2)	2-OH, 2*	2*	1, 2*, 3*	2-OH, 2*, 3*, GlyI-NH, GlyI/II-2', GlyII-NH, AHOPA-3
	2*	84.9	4.54, dd (1)	2, 3* / 2-OH, 3*-NH, 4,4*'	2, 3*	2, 3*, 5*-CO	2, 3*, 3*-NH, 4*,4* ', GlyI-NH, GlyI/II-2', GlyII-NH, AHOPA-3
	3*	44.5	4.4, dddd (3.0, 3.0, 7.3, 9.9)	2*, 3*-NH, 4,4*'	2*, 4*	-	2, 2*, 3*-NH, 4*,4* ', GlyII-NH
	3*-NH	-252.2	8.13, d (7.3)	3* / 2*, 4*, 4* '	3*	2*, 3*, AHOPA-1	2*, 3*, 4*, 4*', AHOPA-2, 3, 3-NH
	4*	35.4	2.24, dd (3.0, 17.8)	3*, 4* ' / 2*, 3*-NH	3*	3*, 5*-CO	3*, 3*-NH, 4*'
	4* '		2.72, dd (9.9, 17.8)	3*, 4* / 2*, 3*-NH	3*	2*, 3*, 5*-CO	3*, 3*-NH, 4*
	5*-CO	175.6					
AHOPA	CO	171.9					
	2	71.1	3.73, dd (2.1, 5,49)	2-OH, 3 / 3-NH, 4,4'	-	1, 3, 4	3, 3-NH, AOFHA-3*-NH, 4*
	2-OH		5.57	2	2	1, 2, 3, 5	2, 3, 4,4', AOFHA-3*-NH
	3	51.1	3.93, m (2.1, 6, 8.8, 9)	2, 3-NH, 4,4' / 2-OH, (6, 7, 8)	4	1, 4, 5, GlyI-1	2, 5, 3-NH, AOFHA-3*-NH, 4*
	3-NH	-256.1	7.54, d (8.8)	3 / 2, 4,4', 5, (6, 7, 8)	3	3, GlyI-1	3, 4,4', GlyI-2, GlyI/II-2', AOFHA-3*-NH
	4	32.6	2.16	3, 4', 5 / 2, 3-NH, (6, 7, 8)	3, 5	2, 3, 5, 6	3, 3-NH, 4', (6, 7, 8)
	4'		2.3	3, 4, 5 / 2, 3-NH, (6, 7, 8)	3, 5	2, 3, 5, 6	2, 3, 3-NH, 4, (6, 7, 8)
	5	129.9	5.55	(6, 7, 8)	4, 6	7	2, 3, 4,4', (6, 7, 8)
	6	132.1	6.07[e]			7	
	7	130.5	6.07[e]			6	
	8	131.2	6.06[e]			(7, 9)	
	9	130.5	6.03	(6, 7, 8)	10	7	10, (11, 12)
	10	133.4	5.64, dt (14.0)	9, 11	9, 11	8, 11	(6, 7, 8), 11, 12
	11	31.6	2.1	10 / (6, 7, 8, 9)	10, 12	10, 12	9, (14, 15)
	12	31.3	2.08	13 / (14, 15)	11, 13	11, 13	(14, 15)
	13	130.9	5.51, m (6.4, 14)	12, 14	12	12, 15	(11, 12), (14, 15)

14	130.6	5.96	13	13	12, 16, 17	(11, 12), 16
15	129.0	5.94	16	16	17	(11, 12), 16
16	133.7	5.57	15, 17	15, 17	14, 17, 18	(14, 15), 17
17	24.5	1.98, dq (7,3)	16, 18 / (14, 15)	16, 18	15, 16, 18	(14, 15), 16, 18
18	13.2	0.91, t (7.3)	17 / 16, (14, 15)	17	16, 17	3-NH, (6, 7, 8), 17

[a]Relative to NO^{3-}; [b]δH relative to TMS and *J* values of AHOPA methine protons 5...16 between 13...15 Hz measured from HSQC ; [c]Correlations in COSY / only in TOCSY

[d]HMBC and H2BC correlations are from the proton(s) stated to the indicated carbon.

[e] and ( ) = overlapping signal(s), [f]Broad signals, couplings smaller than the linewidth.

AOFHA = (3-amino-5-oxotetrahydrofuran-2-yl)(hydroxy)acetic acid

AHOPA = (5*E* ,7*E* ,9*E* ,13*E* ,15*E*)-3-amino-2-hydroxyoctadeca-5,7,9,13,15-pentaenoic acid

AHOTA = (5*E* ,7*E* ,9*E* ,13*E*)-3-amino-2-hydroxyoctadeca-5,7,9,15-tetraenoic acid
