# Supplementary material for: Anabaenolysins, Novel Cytolytic Lipopeptides from Benthic Anabaena Cyanobacteria
Source: PLoS One. 2012 Jul 19;7(7):e41222. doi: 10.1371/journal.pone.0041222 (PMC3400675; doi:10.1371/journal.pone.0041222)
Supplement: Table S2 — Product ion assignments from the fragmentation of the unlabeled and 15N-labeled protonated anabaenolysin A. (PDF) [file pone.0041222.s016.pdf]

Table S2. Product ion assignments from the fragmentation of the unlabeled and ^{15}N-labeled protonated anabaenolysin A. Accurate mass measurements were made with LTQ Orbitrap (ThermoFisher Scientific) using nanospray infusion of the purified 1 and both collision induced dissociation (CID) and higher energy collisional dissociation (HCD) to produce product ions.

Ion trap measurement			Accurate mass measurement			
Ion (m/z)[a] ^{14}N	^{15}N	Number of N	Experimental mass	Calculated mass	Error (ppm)	Assigned ion structure without H^+
559	563	4	559,27600	559,27623	-0,42	M
542	545	3				M-NH_3
541	545	4	541,26428	541,26567	-2,57	M-H_2O
531	535	4	531,28009	531,28132	-2,32	M-CO
524	527	3				M-(H_2O+NH_3)
514	517	3				M-(CO+NH_3)
513	517	4	513,26959	513,27076	-2,27	M-(CO+H_2O)
497	499	2				M-(CO+2NH_3)
496	499	3				M-(CO+H_2O+NH_3)
502	505	3				M-Gly
485	487	2	485,22717	485,22822	-2,17	M-(Gly+NH_3)
467	469	2				M-(Gly+H_2O+NH_3)
465	469	4	465,19702	465,19798	-2,07	M-C_7H_{10}
457	459	2	457,23315	457,23331	-0,35	M-(Gly+CO+NH_3)
439	441	2				M-(Gly+CO+H_2O+NH_3)
417	419	2	417,23834	417,23839	-0,13	M-(Gly–Gly+CO)
411	415	4	411,15030	411,15103	-1,79	M-$C_{11}H_{16}$
397	401	4	397,13531	397,13538	-0,19	M-$C_{12}H_{18}$
383	387	4				M-($C_{11}H_{16}$+CO)
369	373	4	369,14041	369,14047	-0,16	M-($C_{12}H_{18}$+CO)
357	359	2				M-(AOFHA+CO+NH_3)
357	361	4				M-$C_{15}H_{22}$
340	343	3				M-($C_{12}H_{18}$+CO+29)
312			312,11896	312,11901	-0,15	M-($C_{12}H_{18}$+Gly+CO)
300	301	1				M-(Gly–AOFHA+CO+NH_3)
300	303	3				M-($C_{15}H_{22}$+Gly)
289	293	4				M-(AHOPA+NH_3)
272	275	3	272,08771	272,08771	0,01	M-AHOPA
270	271	1				AHOPA-(2H_2O)
260	261	1	260,20087	260,20089	-0,07	M-(Gly–Gly–AOFHA+CO)
253	253	0	253,15871	253,15869	0,08	AHOPA-(2H_2O+NH_3)
244	247	3	244,09285	244,09279	0,23	M-(AHOPA+CO)
242	243	1				M-(Gly–Gly–AOFHA+CO+H_2O)
230	231	1	230,19006	230,19033	-1,15	$C_{16}H_{23}N$ from AHOPA
225	225	0	225,16383	225,16378	0,24	AHOPA-(CO+2H_2O+NH_3)
215	217	2	215,06630	215,06624	0,26	M-(AHOPA–Gly)

[a] = In ^{14}N column are product ions from unlabelled Abl-A and in ^{15}N column are product ions from ^{15}N labelled Abl-A.
