# Supplementary material for: Anabaenolysins, Novel Cytolytic Lipopeptides from Benthic Anabaena Cyanobacteria
Source: PLoS One. 2012 Jul 19;7(7):e41222. doi: 10.1371/journal.pone.0041222 (PMC3400675; doi:10.1371/journal.pone.0041222)
Supplement: Table S3 — 1H, 13C and 15N NMR spectral data for anabaenolysin B (2) in DMSO-d6. (PDF) [file pone.0041222.s017.pdf]

Table S3. ^{1}H, ^{13}C and ^{15}N NMR spectral data for anabaenolysin B (major variant) in [D6]DMSO.

	Anabaenolysin B			
**Substructure**	**C/H no**	**δC**	**δH[b]**	**COSY/TOCSY[c]**
Gly I	CO	169.6		
	2	43.5	3,35	(2'), NH
	2'		3,85	2, NH
	NH		7,70	2,2'
Gly II	CO	170.4		
	2	43.3	3,55	(2'), NH
	2'		3,88	2, NH
	NH		8,77	2,2'
AOFHA	CO	171.3		
	2-OH		5,82	2
	2	71.6	4,26	2-OH, 2*
	2*	86.4	4,62	2, 3* / 2-OH, 3*-NH, 4,4*'
	3*	45.7	4,47	2*, 3*-NH, 4,4*'
	3*-NH		8.27	3* / 2*, 4*, 4* '
	4*	36.3	2,28	3*, 4* ' / 2*, 3*-NH
	4* '		2,77	3*, 4* / 2*, 3*-NH
	5*-CO	175.1		
AHOTA	CO	172.4		
	2	72.3	3,79	2-OH, 3 / 3-NH, 4,4'
	2-OH		5,56	2
	3	52.7	3,99	2, 3-NH, 4,4' / 2-OH, (6, 7, 8)
	3-NH		7,60	3 / 2, 4,4', 5, (6, 7, 8)
	4	33.9	2,20	3, 4', 5 / 2, 3-NH, (6, 7, 8)
	4'		2,34	3, 4, 5 / 2, 3-NH, (6, 7, 8)
	5	131.1	5,58	(6, 7, 8, 9)
	6	130.8	6,07	
	7	131.9	6,07	
	8	133.0	6,11	
	9	130.2	6,07	
	10	139.8	5,79	
	11	33.1	2,05	
	12	33.1	2,05	
	13	131.1	5,58	
	14	135.9	5,68	15 / 13, 16
	15	33.1	2,05	14, 16 / 17
	16	33.0	1,35	15, 17 / 14, 18
	17	23.0	1,27	16, 18 / 15
	18	14.2	0,87	17 / 16

[a]Relative to NO^{3-}; [b]Relative to TMS; [c]Correlations after / only in TOCSY

[d]HMBC correlations are from the proton(s) stated to the indicated carbon.

( ) = Fused signal(s)

AOFHA = (3-amino-5-oxotetrahydrofuran-2-yl)(hydroxy)acetic acid

AHOTA = (5*E* ,7*E* ,9*E* ,13*E*)-3-amino-2-hydroxyoctadeca-5,7,9,15-tetraenoic acid
