# Supplementary material for: Anabaenolysins, Novel Cytolytic Lipopeptides from Benthic Anabaena Cyanobacteria
Source: PLoS One. 2012 Jul 19;7(7):e41222. doi: 10.1371/journal.pone.0041222 (PMC3400675; doi:10.1371/journal.pone.0041222)
Supplement: Figure S10 — Structure of Anabaenolysin A with COSY correlations (bold lines) linking germinal and vicinal protons together and 13C HMBC (arrows) correlations linking substructures to each other. (PDF) [file pone.0041222.s010.pdf]

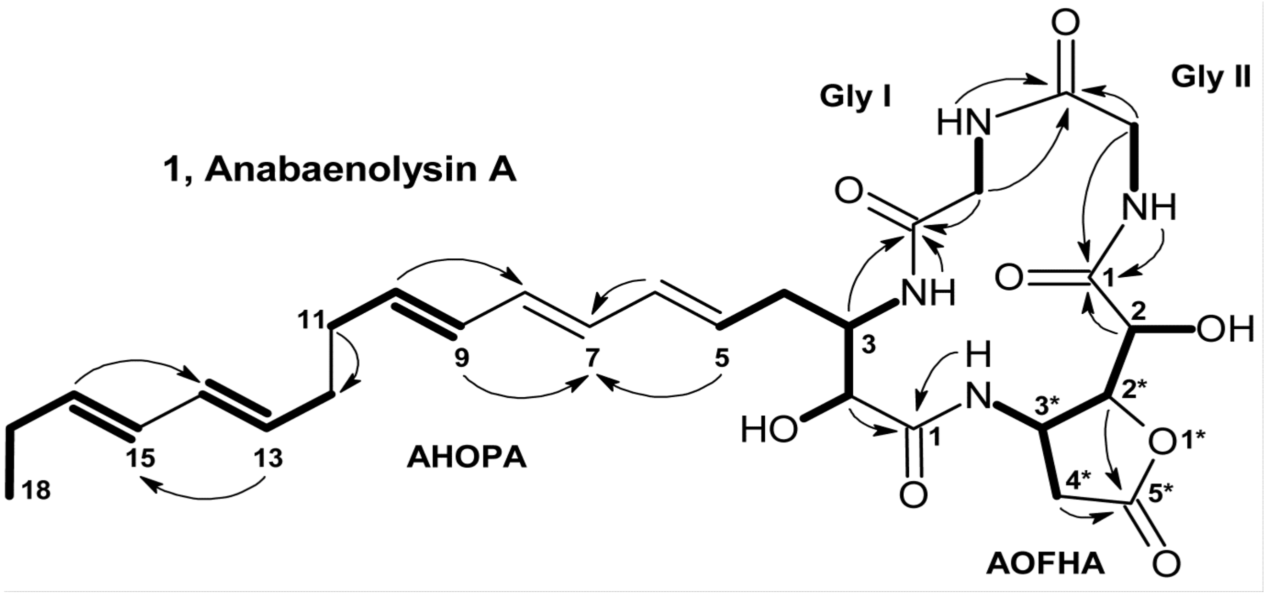

Figure S10. Structure of Anabaenolysin A with COSY correlations (bold lines) linking atoms next to each other together and 13C HMBC (arrows) correlations linking substructures to each other.
