# Supplementary material for: Anabaenolysins, Novel Cytolytic Lipopeptides from Benthic Anabaena Cyanobacteria
Source: PLoS One. 2012 Jul 19;7(7):e41222. doi: 10.1371/journal.pone.0041222 (PMC3400675; doi:10.1371/journal.pone.0041222)
Supplement: Table S4 — Anabaenolysin variants found in the Anabaena strain XPORK 15F. Identification based on UV-spectra showing the triene specific absorption pattern, ion mass [MH]+, product ion spectra and retention time (Rt). Product ions presented characterise the differences in the structures. (PDF) [file pone.0041222.s018.pdf]

Table S4. Anabaenolysin variants found in the *Anabaena* strain XPORK 15F. Identification based on UV-spectra showing the triene specific absorption pattern, ion mass $[MH]^+$, product ion spectra and retention time (R_t). Product ions presented characterise the differences in the structures.

Variant No	R_t (min)	MH^+ (m/z)	Relative area (%)	$M-C_{11}H_{16}$	$M-C_{12}H_{18}$	M-FA[b]	FA-($2H_2O$)	FA-CO	FA-($2H_2O+NH_3$)	FA-($CO+H_2O$)	FA-NH_2	FA-($CO+2H_2O+NH_3$)
1, Abl A	23.5	559	78	411	397	272	270	260	253	242	230	225
2, Abl B	23.9	561	7	411	397	272	272	262	255	244	232	227
3	21.7	531	2	411	397	272	242	232	225	214	202	197
4[c]	21.7	553	2									
5	22.4	555	1	411	397	272	266	nd[d]	251	240	228	nd
6	22.5	545	1	411	397	272	256	246	239	228	216	211
7	22.9	557	2	411	397	272	268	258	251	240	228	223
8	23.3	573	2	425	411	286	270	260	253	242	230	225
9	24.4	573	2	411	397	272	284	274	267	256	244	239
10	24.7	563	3	411	397	272	274	264	257	246	234	229

a: Calculated from the MH^+ signals, b: FA = unsaturated hydroxyamino-fatty acid
c: Ion assignment not done because of nontypical fragmentation, d: nd = not detected
